# The relationship between 3,4‐methylenedioxymethamphetamine (MDMA) use in young adulthood and anxiety or depressive disorders in the mid‐30s: Findings from the Victorian Adolescent Health Cohort Study

**DOI:** 10.1111/add.70173

**Published:** 2025-08-28

**Authors:** Zachary Bryant, Kirsten Morley, Jessica A. Kerr, Craig A. Olsson, Tim Slade

**Affiliations:** ^1^ The Matilda Centre for Research in Mental Health and Substance Use, Faculty of Medicine and Health University of Sydney Sydney Australia; ^2^ Discipline of Addiction Medicine, Central Clinical School, Faculty of Medicine and Health University of Sydney Sydney Australia; ^3^ Department of Psychological Medicine University of Otago Christchurch New Zealand; ^4^ Murdoch Children's Research Institute Parkville Australia; ^5^ Department of Paediatrics University of Melbourne Parkville Australia; ^6^ Deakin University, Faculty of Health School of Psychology, SEED Centre for Lifespan Research Burwood Australia

**Keywords:** 3,4‐methylenedioxymethamphetamine, anxiety, depression, MDMA, mental health, mood, psychedelics, stimulants, substance use

## Abstract

**Background and aims:**

MDMA (3,4‐methylenedioxymethamphetamine or “Ecstasy”) is the fourth‐most used illicit substance globally. While previous research found links between MDMA use and mental health outcomes, the direction and nature of this relationship remain unclear. This study assessed whether MDMA use in early adulthood increases the risk of anxiety or depression in mid‐30s.

**Design:**

A longitudinal, population‐based study using doubly robust inverse probability treatment weighted regression analysis, a contemporary confounder adjustment technique, to examine the relationship between MDMA use in early adulthood (age 20–29) and subsequent anxiety or depression at age 35.

**Setting:**

Victoria, Australia.

**Participants:**

Data were drawn from the Victorian Adolescent Health Cohort Study (VAHCS), which began in 1992 with a statewide representative sample of 1943 Year 9 students (aged 14–15) from 44 Victorian schools. This paper uses data collected from wave 2 to wave 10 (ages 15–35).

**Measurements:**

Across waves 7–9 (ages 20–29), MDMA use was categorised as any use, persistent use (none, one wave, two or more waves) and frequent use (none, infrequent, frequent). Wave 10 (age 35) outcomes were 12‐month diagnoses of major depressive disorder and anxiety disorders assessed using the Composite International Diagnostic Interview (CIDI).

**Findings:**

There was little evidence linking any pattern of MDMA use in early adulthood with depressive disorders by the mid‐30s; however, compared with non‐MDMA users, the adjusted odds of an anxiety disorder were higher in those who reported past 12‐month MDMA use [odds ratio (OR) = 1.73, 95% confidence interval (CI) = 1.12–2.68), persistent MDMA use at two or more waves (OR = 2.05, 95% CI = 1.07–3.94), as well as infrequent (OR = 2.11, 95% CI = 1.14–3.92) and frequent MDMA use (OR = 2.56, 95% CI = 1.15–5.71).

**Conclusions:**

MDMA use (3,4‐methylenedioxymethamphetamine or “Ecstasy”) in early adulthood appears to be associated with increased odds of anxiety disorders but not depressive disorder by the mid‐30s.

## INTRODUCTION

3–4 methylenedioxymethamphetamine or MDMA (‘Ecstasy’) is the fourth most used illicit substance globally [1]. Recent findings reveal that Australians are using MDMA at one of the highest rates in the world with a past year prevalence of 2.8%, threefold higher than the global average [1]. Young adults (20–29 years) are using at the highest rate of any age group, with a past year prevalence of 7.5% [2]. MDMA can elicit effects of euphoria, mood elevation and increased sociability [3]. However, negative effects such as increased heart rate, nausea, vomiting, sleep disturbances and low mood and energy in the days following use are also frequently reported [3]. In the brain, MDMA modulates the release of serotonin, dopamine and norepinephrine, which are responsible for the distinct psychotropic effects of MDMA [4, 5]. Serotonergic neurotransmission in particular has been implicated in a wide range mood and behaviour outcomes including emotion regulation, sleep–wake cycles, movement, appetite, aggression, impulsivity and cognition [6, 7, 8].

In population surveillance data, anxiety and depression frequently found to co‐occur with MDMA use. For examples, Australian national survey data found that 20% and 16% of people who used MDMA also reported being diagnosed with an anxiety disorder or depression, respectively, in the past year [2]. This is compared to 13% for anxiety disorder and 13% for depression diagnosis in those who do not report using MDMA in the past 12 months. Polysubstance use is also common in people who use MDMA, with recent estimates finding that 67.6% of people who reported recent MDMA use also reported using at least one other illicit substance at the same time [2]. Several cross‐sectional studies have found positive associations between MDMA use, anxiety and depression symptoms [9, 10, 11], although few studies have examined this relationship longitudinally, which inherently limits the ability to draw any conclusions regarding directionality.

Of the longitudinal investigations to date, Lieb *et al*. [12] found that among a large representative community sample (*n* = 2462), 88% who reported using MDMA and met the criteria for a DSM‐IV mental disorder, reported the age of onset of their disorder as before MDMA initiation, inferring mental health as temporally prior. However, when compared to non‐users there was no evidence of a relationship between mental disorders and MDMA use. Furthermore, the longitudinal investigations by Daumann *et al*. [13] and George *et al*. [14] found higher self‐report symptoms of depression in young people who regularly use MDMA compared to non‐users. However, after adjusting for prior mental health and other substance use, MDMA use was not associated with long‐term depression diagnosis. Likewise, Brière *et al*. [15] found MDMA use in grade 10 to be associated with increased likelihood of elevated depression symptoms when followed up in grade 11, however, this relationship was no longer statistically significant after controlling for important confounds including pre‐existing mental health, other substance use and environmental confounders including family and peer factors.

A central limitation is the lack of prospective data on the long‐term effects of MDMA use on anxiety and depression collected within longitudinal study designs. Such designs are essential for understanding temporal sequencing between exposure and outcome. Definitional imprecision, insufficient attention to confounding and underpowered studies also continues to limit progress. For example, Lieb *et al*. [12] pooled MDMA and related drugs such as amphetamines together, making it difficult to identify a substance‐specific effect. Limited confounder adjustment for pre‐existing mental health and other substance use also reduced confidence in making causal interpretations in this study. Daumann *et al*. [13] reported on a relatively small clinical sample (*n* = 38 at follow‐up) making it difficult to draw inferences about the general population. Further, there were baseline differences in depression between comparator groups, likely confounding the relationship between exposure and outcome. George *et al*. [14] pooled participants who reported MDMA use across a wide range of frequencies (from every day to once or twice a year), therefore, failing to consider the effect of varying exposure levels. Additionally, George *et al*. [14] measured the exposure outside of the peak period of initiation where MDMA use tends to subside. Brière *et al*. [15] measured MDMA use before the typical age of initiation, therefore, failing to capture exposure when it is most likely to occur (i.e. young adulthood). Further, Brière *et al*. [15] measured confounding variables at the same timepoint as the exposure variable, meaning that their temporal sequencing is difficult to establish, which in turn may bias effect estimates.

In addition to the methodological limitations of previous research, no studies have implemented contemporary statistical approaches to enhancing causal inference when investigating the relationship between MDMA use and mental health outcomes such as clinically significant anxiety or depressive symptoms, diagnostic status or severity levels. Recent advances in statistical analysis of observational studies have allowed researchers to better assess causal relationships outside the randomised controlled trial setting [16]. In observational studies, a lack of randomisation can introduce systematic differences in participant characteristics among exposure groups. This may result in confounder bias, where shared causes of both exposure and outcome distort the observed relationship, leading to either an over or underestimation of the true effect [16, 17]. Addressing confounder bias is crucial for accurately estimating causal relationships from observational data. Inverse probability treatment weighting (IPTW) is a confounder adjustment method that uses propensity scores to balance participants across exposure groups on important confounding variables [16, 18]. The application of these weights creates a pseudo‐population in which the relationships between confounders and exposure are independent, and so any differences in outcome can be attributed to exposure alone.

Taken together, in this study we aimed to investigate the relationship between early adulthood MDMA use and subsequent anxiety or depression disorders using repeated measures data from one of Australia's longest running studies of health and development, The Victorian Adolescent Health Cohort Study (est, 1992). We used prospective data collected from the early 20s to the mid‐30s across multiple follow‐up occasions encompassing the age of initiation and the period of peak use. We aimed to adequately control for important pre‐exposure confounding factors, using methods to improve causal inference.

Specifically, our aims were to (1) examine the relationship between exposure to MDMA use in early adulthood and a depression diagnosis by the mid‐30s; (2) examine the relationship between MDMA use and any anxiety disorder diagnosis by the mid‐30s; and (3) examine the effect of three separate measures of MDMA exposure on the above outcomes. Based on prior evidence we hypothesised that: [hypothesis 1 (H1)] individuals who reported any MDMA use during young adulthood would not increase odds of receiving a later diagnosis of anxiety or depression, compared to non‐users after controlling for important confounds; (H2) people who use MDMA persistently use across multiple waves would be associated with a greater cumulative risk of anxiety or depression diagnosis relative to intermittent or non‐use; and (H3) frequent MDMA use, when compared to no use would be associated with greatest odds of subsequent anxiety or depression diagnosis consistent with a dose–response relationship.

## METHODS

### Sample

Data for the current study were derived from the Victorian Adolescent Health Cohort Study (VAHCS), a long‐standing population‐based longitudinal study initiated in 1992 as a state‐wide representative sample of Year 9 students (14–15 years old) from secondary schools in Victoria (Table 1). Participants were followed up 11 times across three decades, with assessments during adolescence (ages 14–17), young adulthood (approximate ages 21 and 24), and adulthood (approximate ages 29, 35, and 41). The most recent wave (wave 11) conducted in 2019 to 2021, in which 73% of the cohort was retained. Further details on the sample can be found in the Supporting information.

**TABLE 1:** VAHCS study timeline.

	First sample	Second sample									
Wave	1	2	3	4	5	6	7	8	9	10	11
Year	1992	1992	1993	1994	1994	1995	1998	2001–03	2006–08	2012–2014	2019–2021
Mean age	14.9	15.5	15.9	16.4	16.8	17.4	20.7	24.1	29.1	35.1	42.6
*n*	898	1727	1697	1628	1575	1530	1601	1520	1501	1443	1428

Abbreviation: VAHCS, Victorian Adolescent Health Cohort Study.

### Measures

#### Demographics

Age (years; waves 7 and 10) was measured as a continuous variable. Sex assigned at birth was measured as a binary variable 0 = female and 1 = male. Socio‐economic status was measured at wave 3 using the Index of Relative Socio‐economic Disadvantage (IRSD) and is scored across 5 quintiles with Q1 = most disadvantage to Q5 = least disadvantaged [19].

#### Exposures

##### MDMA use in early adulthood (20–29 years)

Any MDMA use in the past 12 months and frequency of MDMA use in the past 12 months was measured at waves 7 and 9 (20–29 years). These waves represented the peak period for MDMA use (see Supporting information for prevalence of MDMA use across waves). Researchers derived three exposure variables from the exposure period, and any use over the exposure period coded 0 = no use and 1 = any use. A variable for any past 12‐month frequency of MDMA use over the exposure period coded as 0 = no use, 1 = infrequent/experimental (less than monthly) use and 2 = frequent use (greater than monthly). A persistent use variable was created for participants who had used MDMA across waves coded 0 = use at no waves, 1 = exposure at 1 wave, 2 = exposure at 2 or more waves throughout the exposure period. There were 108 participants who had exposure information on any MDMA use in the past 12 months at wave 8 of the study, however, information on frequency of MDMA use was not collected at this wave. Therefore, their data were excluded from regression models with frequency of MDMA use as exposure.

#### Outcomes

##### Mental health at age 35 years

The outcome measures were any Composite International Diagnostic Interview (CIDI) major depressive disorder diagnosis and any CIDI anxiety disorder diagnosis (any generalised anxiety disorder, social phobia, panic disorder and agoraphobia) in the past 12 months [20]. Despite data being available at wave 11, wave 10 was chosen as the outcome measurement timepoint because this is closest in time after the exposure window. Both disorders were defined according to the International Classification of Diseases 10th revision (ICD‐10), with depressive disorder assessed using the CIDI‐Auto and anxiety disorder using the CIDI‐Short Form [21, 22]. Both instruments have been validated for use in population‐based surveys and are recommended by the World Health Organization for the standardized assessment of ICD‐10 mental disorders [23, 24, 25]. These were measured at wave 10 and coded 0 = no diagnosis and 1 = diagnosis if participants met the specific criteria for depressive disorder or anxiety disorder.

### Potential confounders

#### Confounder selection

To mitigate bias and follow best practice in generating IPT weights, all confounders were measured pre‐exposure and included variables known to be associated with both the exposure and outcome, or with the outcome alone, using the ‘disjunctive cause criterion’ [16, 26, 27, 28]. This approach supports conditional exchangeability and improves covariate balance between exposure groups so that following weighting (detailed below) groups only differ on exposure information. Specifically, covariates used to balance across exposure groups included sex, socio‐economic status, parental divorce, general health, prior substance use (smoking, binge drinking, cannabis, amphetamines and other drugs), peer and parental substance use, impulsivity, anxiety and depression symptoms [29, 30, 31, 32, 33].

#### General health

##### General health

Self‐report general health at wave 6 (18 years), was measured as a categorical variable coded as 0 = poor, 1 = fair, 2 = good and 3 = excellent. This is a single item scale that was developed for the VAHCS study.

##### Adolescent mental health

Pre‐exposure anxiety and depression scores at wave 6 (18 years) were, measured as continuous variables using the Clinical Interview Schedule (CIS‐R), a validated self‐report instrument with strong internal consistency and reliability for detecting common mental disorders, with scores ranging from 0 to 4 [23, 34, 35]. Adolescent impulsivity was measured at wave 2 (14 years) as a continuous variable with scores ranging from 0 indicating low impulsivity to 16 indicating high impulsivity [36].

##### Other substance use

Past 12‐month tobacco use was measured at wave 6 (18 years) as a binary variable coded 0 = no use and 1 = use. Alcohol use was measured as a binary any high‐risk drinking (> = 5 standard drinks on at least 1 day) in the past 12 months coded 0 = no high risk drinking and 1 = high risk drinking. In Australia, 1 standard drink contains 10 mg of alcohol. Past 12‐month cannabis use, amphetamine use, and other drug use were measured as binary variables coded 0 = no use and 1 = any use. Peer alcohol use was measured as a 3‐level categorical variable coded 0 = no peers drink alcohol, 1 = some peers drink alcohol and 2 = most peers drink alcohol.

##### Parent marital status, education and substance use

Parental divorce was measured at wave 6 as a binary variable coded 0 = no and 1 = yes. Parental education was measured at wave 6 as a binary variable coded 0 = at least 1 parent completing grade 12 and 1 = neither parent completed grade 12. Parental alcohol and tobacco use were measured at wave 6 (18 years) as binary variables coded as 0 = never/infrequent use and 1 = frequent/everyday use.

### Statistical analysis

To examine the strength and independence of associations between MDMA use and anxiety or depression, unadjusted and adjusted ORs of (1) any MDMA use on; (2) frequency of MDMA use; and (3) persistent MDMA use on any anxiety or depression diagnosis were estimated using traditional logistic regression models.

### Weights

To reduce bias from measured confounding in estimating the effect of the exposure on the outcome, IPT weights were applied to the sample by weighting participants by the inverse probability of exposure based on values of observed model covariates [27]. Weights were derived for each MDMA exposure variable separately, using the *weightit* package in R [37]. Final IPT weighted logistic regression models included all covariates to ensure that effect estimates were doubly robust [38]. Extreme weights were handled via truncation at the 95th percentile, whereby adequate covariate balance across exposure groups (as indicated by a maximum standardised mean difference between groups of ~0.1) was achieved while reducing the influence of extreme values (see Supporting information) [16, 39]. E‐values, which represent the minimum strength of association that an unmeasured confounder would need to have with both the exposure and the outcome, beyond measured covariates, to fully explain away the observed association were calculated in further sensitivity analyses (see Supporting information for details) [40].

### Multiple imputation

Data were assumed to be missing at random and were handled using multiple imputation by chained equations, using the *mice* package in R [41, 42]. There were approximately 20% incomplete cases, therefore, 20 datasets were imputed using all analytic model covariates. Only participants with outcome and exposure information were included in the final analyses, which resulted in a total analytic sample of 1329 participants.

Three separate models were run for each exposure‐outcome combination: (1) unadjusted models; (2) imputed and adjusted models; and (3) doubly robust IPTW models. Variance inflation factors (VIFs) were used to assess collinearity between variables in the multivariable models, with a VIF >10 indicating high collinearity. All analyses were completed using R statistical software version 4.3.2. As the analysis plan was not pre‐registered, any results should be considered exploratory.

## RESULTS

### Sample characteristics

Table 2 below shows the descriptive statistics of the analysed sample.

**TABLE 2:** Participant characteristics.

Participant characteristics	*n* (1329) or mean	% or SD
Sex		
Female	727	55%
Male	602	45%
Age wave 7		
Mean (SD)	20.7	0.4
Age wave 10		
Mean (SD)	35	0.5
Nationality		
Born in Australia	1136	85%
Born outside of Australia	152	11%
Missing	41	4%
Education		
Did not complete grade 12	161	12%
Completed grade 12	1116	84%
Missing	52	4%
Employed fulltime at wave 10		
No	506	38%
Yes	823	62%
Any anxiety disorder diagnosis wave 10		
No	1178	89%
Yes	151	11%
Depression diagnosis wave 10		
No	1176	88%
Yes	153	12%
Any MDMA use		
No	994	75%
Yes	335	25%
Any persistent MDMA use		
No use	994	75%
Use at 1 wave	201	15%
Use at 2 or more waves	134	10%
Any frequent MDMA use		
No use	994	75%
Infrequent/experimental use	166	12%
Frequent use	61	5%
Missing	108	8%

Abbreviation: MDMA, 3,4‐methylenedioxymethamphetamine.

### Depression

In unadjusted models, we found limited evidence of an association between MDMA use and later diagnosis of major depressive disorder in the mid‐30s. This finding was consistent across different conceptualisations of MDMA use (any use, frequent use and persistent use). Pattern of findings were similar across the three models (see Tables 3, 4, 5).

**TABLE 3:** Regression models for the relationship between any MDMA use and depression.

Characteristic	Unadjusted model (*n* = 1329)			Imputed and adjusted model (*n* = 1329)			Doubly robust IPTW model (*n* = 1329)		
	OR	95% CI	*P*	OR	95% CI	*P*	OR	95% CI	*P*
Any MDMA use									
No	–	–	–	–	–	–	–	–	–
Yes	1.36	0.93–1.95	0.1	1.21	0.80–1.84	0.4	1.12	0.73–1.73	0.6
Sex									
Female				–	–	–	–	–	–
Male				0.98	0.67–1.44	>0.9	1.12	0.72–1.74	0.6
Nationality									
Born in Aus				–	–	–	–	–	–
Born outside of Aus				0.64	0.34–1.22	0.2	0.72	0.33–1.56	0.4
Socio‐economic disadvantage quintiles									
Q1 (most disadvantaged)				–	–	–	–	–	–
Q2				0.69	0.35–1.35	0.3	0.76	0.33–1.75	0.5
Q3				0.58	0.28–1.19	0.13	0.79	0.32–1.96	0.6
Q4				0.65	0.35–1.19	0.2	0.64	0.30–1.37	0.2
Q5 (least disadvantaged)				0.77	0.45–1.33	0.3	0.66	0.35–1.27	0.2
Parental divorce									
No				–	–	–	–	–	–
Yes				1.46	0.96–2.21	0.08	1.52	0.92–2.51	0.1
General health									
Excellent				–	–	–	–	–	–
Good				1.18	0.70–2.00	0.5	1.35	0.75–2.43	0.3
Fair				1.77	0.96–3.26	0.07	2.06	1.03–4.10	**0.04**
Poor				2.14	0.69–6.61	0.2	3.41	0.96–12.1	0.06
Any smoking									
No				–	–	–	–	–	–
Yes				1.18	0.74–1.88	0.5	1.18	0.67–2.05	0.6
Any binge drinking									
No				–	–	–	–	–	–
Yes				0.94	0.58–1.52	0.8	0.97	0.56–1.70	>0.9
Any cannabis use									
No				–	–	–	–	–	–
Yes				1.02	0.56–1.86	>0.9	1.05	0.56–1.98	0.9
Any amphetamine use									
No				–	–	–	–	–	–
Yes				0.57	0.17–1.93	0.4	0.47	0.13–1.80	0.3
Any other drug use									
No				–	–	–	–	–	–
Yes				1.9	0.76–4.75	0.2	1.83	0.67–5.01	0.2
Peer substance use									
None				–	–	–	–	–	–
Some				1.21	0.77–1.90	0.4	1.09	0.64–1.88	0.7
Most				0.97	0.43–2.22	>0.9	0.83	0.34–2.03	0.7
Parental smoking									
Never/infrequent				–	–	–	–	–	–
Frequent/everyday				1.11	0.74–1.67	0.6	1.19	0.74–1.94	0.5
Parental alcohol use									
Never/infrequent				–	–	–	–	–	–
Frequent/everyday				0.74	0.47–1.15	0.2	0.86	0.52–1.41	0.5
Anxiety score at wave 6				1.09	0.83–1.43	0.5	1	0.70–1.42	>0.9
Depression score at wave 6				1.1	0.86–1.40	0.4	1.13	0.81–1.56	0.5
Impulsivity score				1	0.94–1.06	>0.9	1.02	0.95–1.10	0.5

Abbreviations: Aus, Australia; IPTW, inverse probability treatment weighting; MDMA, 3,4‐methylenedioxymethamphetamine.

**TABLE 4:** Regression models for the relationship between persistent MDMA use and depression.

Characteristic	Unadjusted model (*n* = 1329)			Imputed and adjusted model (*n* = 1329)			Doubly robust IPTW model (*n* = 1329)		
	OR	95% CI	*P*	OR	95% CI	*P*	OR	95% CI	*P*
Any persistent MDMA use									
MDMA use no waves	–	–	–	–	–	–	–	–	–
MDMA use 1 wave	1.23	0.76–1.92	0.4	1.1	0.67–1.80	0.7	0.95	0.56–1.61	0.8
MDMA use 2 or more waves	1.56	0.92–2.54	0.09	1.41	0.79–2.50	0.2	1.38	0.74–2.60	0.3
Sex									
Female				–	–	–	–	–	–
Male				0.98	0.67–1.44	>0.9	1.11	0.67–1.83	0.7
Nationality									
Born in Aus				–	–	–	–	–	–
Born outside of Aus				0.64	0.34–1.21	0.2	0.85	0.35–2.05	0.7
Socio‐economic disadvantage quintiles									
Q1 (most disadvantaged)				–	–	–	–	–	–
Q2				0.69	0.35–1.35	0.3	0.78	0.30–2.02	0.6
Q3				0.57	0.28–1.18	0.13	0.82	0.30–2.27	0.7
Q4				0.65	0.35–1.19	0.2	0.6	0.25–1.44	0.3
Q5 (least disadvantaged)				0.77	0.45–1.33	0.3	0.58	0.28–1.23	0.2
Parental divorce									
No				–	–	–	–	–	–
Yes				1.47	0.97–2.24	0.07	1.75	1.0–3.07	0.05
General health									
Excellent				–	–	–	–	–	–
Good				1.17	0.69–1.99	0.6	1.36	0.71–2.60	0.3
Fair				1.76	0.96–3.24	0.07	2.08	0.96–4.51	0.06
Poor				2.11	0.68–6.54	0.2	5.02	1.17–21.6	0.03
Any smoking									
No				–	–	–	–	–	–
Yes				1.17	0.73–1.88	0.5	1.23	0.65–2.33	0.5
Any binge drinking									
No				–	–	–	–	–	–
Yes				0.93	0.58–1.51	0.8	1.02	0.55–1.89	>0.9
Any cannabis use									
No				–	–	–	–	–	–
Yes				1	0.55–1.83	>0.9	0.84	0.40–1.74	0.6
Any amphetamine use									
No				–	–	–	–	–	–
Yes				0.58	0.17–1.94	0.4	0.43	0.10–1.87	0.3
Any other drug use									
No				–	–	–	–	–	–
Yes				1.9	0.76–4.73	0.2	2.33	0.77–7.03	0.13
Peer substance use									
None				–	–	–	–	–	–
Some				1.21	0.77–1.90	0.4	1.05	0.54–2.02	0.9
Most				0.98	0.43–2.22	>0.9	0.95	0.34–2.67	>0.9
Parental smoking									
Never/infrequent				–	–	–	–	–	–
Frequent/everyday				1.12	0.74–1.68	0.6	1.15	0.66–2.00	0.6
Parental alcohol use									
Never/infrequent				–	–	–	–	–	–
Frequent/everyday				0.74	0.47–1.16	0.2	1	0.57–1.75	>0.9
Anxiety score at wave 6				1.09	0.83–1.43	0.5	0.87	0.56–1.35	0.5
Depression score at wave 6				1.1	0.86–1.40	0.5	1.08	0.72–1.62	0.7
Impulsivity score				1	0.94–1.06	>0.9	1.03	0.95–1.12	0.5

Abbreviations: Aus, Australia; IPTW, inverse probability treatment weighting; MDMA, 3,4‐methylenedioxymethamphetamine.

**TABLE 5:** Regression models for the relationship between any frequent MDMA use and depression.

Characteristic	Unadjusted model (*n* = 1221)			Imputed and adjusted model (*n* = 1221)			Doubly robust IPTW model (*n* = 1221)		
	OR	95% CI	*P*	OR	95% CI	*P*	OR	95% CI	*P*
Any frequent MDMA use									
Never	–	–	–	–	–	–	–	–	–
Infrequent/experimental	1.33	0.81–2.13	0.2	0.95	0.68–1.33	0.8	1.04	0.59–1.84	0.9
Frequent	1.63	0.76–3.17	0.2	1.67	0.97–2.91	0.07	1.5	0.62–3.63	0.4
Sex									
Female				–	–	–	–	–	–
Male				1.38	0.99–1.91	0.05	1.19	0.67–2.12	0.6
Nationality									
Born in Aus				–	–	–	–	–	–
Born outside of Aus				2.61	1.53–4.44	<0.001	1.18	0.46–3.02	0.7
Socio‐economic disadvantage quintiles									
Q1 (most disadvantaged)				–	–	–	–	–	–
Q2				0.31	0.17–0.57	<0.001	0.6	0.20–1.78	0.4
Q3				0.55	0.24–1.30	0.2	0.79	0.26–2.40	0.7
Q4				0.48	0.28–0.83	0.009	0.64	0.24–1.70	0.4
Q5 (least disadvantaged)				0.25	0.15–0.43	<0.001	0.54	0.22–1.33	0.2
Parental divorce									
No				–	–	–	–	–	–
Yes				1.95	0.93–4.08	0.07	1.79	0.89–3.58	0.1
General health									
Excellent				–	–	–	–	–	–
Good				2.88	1.60–5.18	<0.001	1.7	0.75–3.85	0.2
Fair				1.72	0.87–3.40	0.12	1.54	0.63–3.75	0.3
Poor				11.9	3.39–41.6	<0.001	9	1.77–45.7	0.008
Any smoking									
No				–	–	–	–	–	–
Yes				1.8	0.90–3.60	0.09	1.57	0.73–3.39	0.3
Any binge drinking									
No				–	–	–	–	–	–
Yes				1.03	0.66–1.60	0.9	0.96	0.44–2.13	>0.9
Any cannabis use									
No				–	–	–	–	–	–
Yes				0.9	0.47–1.73	0.8	0.87	0.39–1.93	0.7
Any amphetamine use									
No				–	–	–	–	–	–
Yes				0.45	0.15–1.35	0.2	0.46	0.08–2.77	0.4
Any other drug use									
No				–	–	–	–	–	–
Yes				2.03	0.62–6.66	0.2	1.88	0.41–8.56	0.4
Peer substance use									
None				–	–	–	–	–	–
Some				1.06	0.51–2.19	0.9	1.1	0.49–2.46	0.8
Most				1.18	0.49–2.87	0.7	1.35	0.46–3.97	0.6
Parental smoking									
Never/infrequent				–	–	–	–	–	–
Frequent/everyday				1.01	0.59–1.72	>0.9	0.9	0.46–1.74	0.8
Parental alcohol use									
Never/Infrequent				–	–	–	–	–	–
Frequent/everyday				1.03	0.59–1.80	>0.9	0.94	0.48–1.83	0.8
Anxiety score at wave 6				0.83	0.53–1.30	0.4	0.93	0.57–1.52	0.8
Depression score at wave 6				1.3	0.94–1.78	0.11	1.25	0.81–1.92	0.3
Impulsivity score				1.03	0.94–1.13	0.5	1.03	0.94–1.14	0.5

Abbreviations: Aus, Australia; IPTW, inverse probability treatment weighting; MDMA, 3,4‐methylenedioxymethamphetamine.

### Anxiety

#### Any MDMA use

In unadjusted models, the odds of any anxiety disorder diagnosis were higher in those reporting any MDMA use compared to non‐use (OR_unadjusted_ = 1.87, CI = 1.30–2.65, *P* = <0.001). This pattern of findings was consistent across all the statistical models with any MDMA use (OR_adjusted_ = 1.73, CI = 1.12–2.68, *P* = 0.01) remaining associated with any anxiety disorder compared to non‐users in the doubly robust IPTW model (see Table 6). The association between any MDMA use (E‐value: point estimate = 2.85; lower CI limit = 1.49) and any anxiety disorder diagnosis was moderately robust to unmeasured confounding.

**TABLE 6:** Regression models for the relationship between any MDMA use and anxiety.

Characteristic	Unadjusted model (*n* = 1329)			Imputed and adjusted model (*n* = 1329)			Doubly robust IPTW model (*n* = 1329)		
	OR	95% CI	*P*	OR	95% CI	*P*	OR	95% CI	*P*
Any MDMA use									
No	–	–	–	–	–	–	–	–	–
Yes	1.87	1.30–2.65	<0.001	1.65	1.08–2.51	0.019	1.73	1.12–2.68	0.01
Sex									
Female				–	–	–	–	–	–
Male				0.6	0.40–0.91	0.015	0.55	0.33–0.92	0.02
Nationality									
Born in Aus				–	–	–	–	–	–
Born outside of Aus				0.91	0.50–1.64	0.7	1.04	0.48–2.23	>0.9
Socio‐economic disadvantage quintiles									
Q1 (most disadvantaged)				–	–	–	–	–	–
Q2				0.59	0.29–1.19	0.14	0.51	0.21–1.25	0.14
Q3				0.27	0.11–0.66	0.004	0.16	0.06–0.44	<0.001
Q4				0.66	0.35–1.22	0.2	0.73	0.34–1.59	0.4
Q5 (least disadvantaged)				0.79	0.45–1.39	0.4	0.89	0.44–1.80	0.7
Parental divorce									
No				–	–	–	–	–	–
Yes				2.2	1.47–3.31	<0.001	1.76	1.07–2.88	0.03
General health									
Excellent				–	–	–	–	–	–
Good				1.16	0.67–2.02	0.6	1.05	0.52–2.11	0.9
Fair				1.54	0.81–2.92	0.2	1.31	0.57–3.01	0.5
Poor				1.46	0.44–4.85	0.5	1.49	0.34–6.58	0.6
Any smoking									
No				–	–	–	–	–	–
Yes				0.88	0.53–1.46	0.6	0.82	0.45–1.49	0.5
Any binge drinking									
No				–	–	–	–	–	–
Yes				1.35	0.79–2.31	0.3	1.31	0.74–2.32	0.4
Any cannabis use									
No				–	–	–	–	–	–
Yes				1.16	0.62–2.14	0.6	1.45	0.74–2.85	0.3
Any amphetamine use									
No				–	–	–	–	–	–
Yes				2.17	0.73–6.43	0.2	1.98	0.59–6.64	0.3
Any other drug use									
No				–	–	–	–	–	–
Yes				0.75	0.26–2.20	0.6	0.74	0.23–2.33	0.6
Peer substance use									
None				–	–	–	–	–	–
Some				1.24	0.76–2.01	0.4	0.98	0.54–1.78	>0.9
Most				1.09	0.48–2.49	0.8	0.72	0.28–1.87	0.5
Parental smoking									
Never/infrequent				–	–	–	–	–	–
Frequent/everyday				0.96	0.62–1.47	0.8	0.99	0.60–1.63	>0.9
Parental alcohol use									
Never/infrequent				–	–	–	–	–	–
Frequent/everyday				0.88	0.56–1.38	0.6	0.9	0.54–1.50	0.7
Anxiety score at wave 6				1.09	0.83–1.43	0.5	1.23	0.91–1.65	0.2
Depression score at wave 6				1.1	0.86–1.40	0.4	1.35	1.04–1.74	**0.02**
Impulsivity score				1	0.94–1.06	>0.9	0.98	0.91–1.05	0.5

Abbreviations: Aus, Australia; IPTW, inverse probability treatment weighting; MDMA, 3,4‐methylenedioxymethamphetamine.

#### Persistent MDMA use

In unadjusted models, the odds of any anxiety disorder diagnosis were higher in those reporting MDMA use at one wave (OR_unadjusted_ = 1.88, CI = 1.22–2.85, *P* = 0.003) and MDMA use at two or more waves (OR_unadjusted_ = 1.84, CI = 1.09–2.99, *P* = 0.02) compared to non‐use. This pattern of findings was relatively consistent across all statistical models. However, only MDMA use at two or more waves (OR_adjusted_ = 2.05, CI = 1.07–3.94, *P* = 0.03) remained associated with any anxiety disorder in the doubly robust IPTW model (see Table 7). The association between MDMA use at two or more waves (E‐value: point estimate = 3.52; lower CI limit = 1.34) and any anxiety disorder diagnosis was moderately robust to unmeasured confounding.

**TABLE 7:** Regression models for the relationship between persistent MDMA use and anxiety.

Characteristic	Unadjusted model (*n* = 1329)			Imputed and adjusted model (*n* = 1329)			Doubly robust IPTW model (*n* = 1329)		
	OR	95% CI	*P*	OR	95% CI	*P*	OR	95% CI	*P*
Any persistent MDMA use									
MDMA use no waves	–	–	–	–	–	–	–	–	–
MDMA use 1 wave	1.88	1.22–2.85	0.003	1.63	1.01–2.62	0.04	1.6	0.96–2.67	0.07
MDMA use 2 or more waves	1.84	1.09–2.99	0.02	1.69	0.94–3.05	0.08	2.05	1.07–3.94	0.03
Sex									
Female				–	–	–	–	–	–
Male				0.6	0.40–0.90	0.02	0.56	0.31–1.00	0.05
Nationality									
Born in Aus				–	–	–	–	–	–
Born outside of Aus				0.9	0.50–1.64	0.7	1.02	0.42–2.46	>0.9
Socio‐economic disadvantage quintiles									
Q1 (most disadvantaged)				–	–	–	–	–	–
Q2				0.59	0.29–1.19	0.14	0.43	0.15–1.25	0.12
Q3				0.27	0.11–0.66	0.004	0.12	0.04–0.36	<0.001
Q4				0.66	0.36–1.22	0.2	0.72	0.29–1.76	0.5
Q5 (least disadvantaged)				0.79	0.45–1.39	0.4	0.86	0.38–1.93	0.7
Parental divorce									
No				–	–	–	–	–	–
Yes				2.21	1.47–3.32	<0.001	1.72	0.97–3.03	0.06
General health									
Excellent				–	–	–	–	–	–
Good				1.16	0.67–2.02	0.6	0.96	0.44–2.12	>0.9
Fair				1.54	0.81–2.92	0.2	1.14	0.45–2.88	0.8
Poor				1.46	0.44–4.85	0.5	1.76	0.37–8.36	0.5
Any smoking									
No				–	–	–	–	–	–
Yes				0.88	0.53–1.46	0.6	0.73	0.37–1.44	0.4
Any binge drinking									
No				–	–	–	–	–	–
Yes				1.35	0.79–2.31	0.3	1.34	0.72–2.52	0.4
Any cannabis use									
No				–	–	–	–	–	–
Yes				1.15	0.62–2.15	0.7	1.41	0.64–3.12	0.4
Any amphetamine use									
No				–	–	–	–	–	–
Yes				2.17	0.73–6.45	0.2	2.29	0.67–7.84	0.2
Any other drug use									
No				–	–	–	–	–	–
Yes				0.75	0.26–2.19	0.6	0.78	0.25–2.40	0.7
Peer substance use									
None				–	–	–	–	–	–
Some				1.24	0.76–2.01	0.4	0.9	0.45–1.80	0.8
Most				1.09	0.48–2.49	0.8	0.81	0.26–2.51	0.7
Parental smoking									
Never/infrequent				–	–	–	–	–	–
Frequent/everyday				0.96	0.62–1.47	0.8	1.01	0.56–1.81	>0.9
Parental alcohol use									
Never/infrequent				–	–	–	–	–	–
Frequent/everyday				0.88	0.56–1.38	0.6	0.92	0.53–1.62	0.8
Anxiety score at wave 6				1.12	0.86–1.46	0.4	1.18	0.86–1.62	0.3
Depression score at wave 6				1.14	0.89–1.47	0.3	1.43	1.07–1.92	0.02
Impulsivity score				0.98	0.92–1.04	0.5	0.99	0.90–1.07	0.7

Abbreviations: Aus, Australia; IPTW, inverse probability treatment weighting; MDMA, 3,4‐methylenedioxymethamphetamine.

#### Any frequent MDMA use

In unadjusted models, the odds of any anxiety disorder diagnosis were higher in those reporting any infrequent/experimental MDMA use (OR_unadjusted_ = 1.98, CI = 1.24–3.09, *P* = 0.004) and any frequent MDMA use (OR_unadjusted_ = 2.54, CI = 1.28–4.73, *P* = 0.005) compared to non‐users. This pattern of findings was consistent across all the statistical models with any infrequent/experimental (OR_adjusted_ = 2.11, CI = 1.14–3.92, *P* = 0.02) and any frequent MDMA use (OR_adjusted_ = 2.56, CI = 1.15–5.71, *P* = 0.02) remaining associated with increased odds of any anxiety disorder compared to non‐users in the doubly robust IPTW model (see Table 8). The association between any infrequent/experimental (E‐value: point estimate = 3.64; lower CI limit = 1.54) and frequent MDMA use (E‐value: point estimate = 4.56; lower CI limit = 1.57) with any anxiety disorder diagnosis were moderately robust to unmeasured confounding.

**TABLE 8:** Regression models for the relationship between any frequent MDMA use and anxiety.

Characteristic	Unadjusted model (*n* = 1329)			Imputed and adjusted model (*n* = 1329)			Doubly robust IPTW model (*n* = 1329)		
	OR	95% CI	*P*	OR	95% CI	*P*	OR	95% CI	*P*
Any frequent MDMA use									
Never	–	–	–	–	–	–	–	–	–
Infrequent/experimental	1.98	1.24–3.09	0.003	2.3	1.63–3.24	<0.001	2.11	1.14–3.92	0.02
Frequent	2.54	1.28–4.73	0.005	2.22	1.13–4.33	0.02	2.56	1.15–5.71	0.02
Sex									
Female				–	–	–	–	–	–
Male				0.49	0.35–0.70	<0.001	0.56	0.30–1.07	0.08
Nationality									
Born in Aus				–	–	–	–	–	–
Born outside of Aus				2.14	0.96–4.78	0.062	1.34	0.50–3.56	0.6
Socio‐economic disadvantage quintiles									
Q1 (most disadvantaged)				–	–	–	–	–	–
Q2				0.31	0.15–0.64	0.002	0.38	0.11–1.31	0.13
Q3				0.06	0.02–0.19	<0.001	0.08	0.02–0.30	<0.001
Q4				1.59	0.93–2.72	0.088	0.98	0.37–2.55	>0.9
Q5 (least disadvantaged)				0.75	0.46–1.21	0.2	0.66	0.27–1.61	0.4
Parental divorce									
No				–	–	–	–	–	–
Yes				2.96	1.71–5.11	<0.001	2.51	1.26–4.97	0.009
General health									
Excellent				–	–	–	–	–	–
Good				1.17	0.53–2.58	0.7	1.51	0.53–4.25	0.4
Fair				1.48	0.55–3.97	0.4	1.72	0.55–5.33	0.3
Poor				3.02	0.81–11.3	0.1	4.11	0.72–23.4	0.11
Any smoking									
No				–	–	–	–	–	–
Yes				0.77	0.38–1.54	0.4	0.74	0.34–1.64	0.5
Any binge drinking									
No				–	–	–	–	–	–
Yes				1.38	0.77–2.48	0.3	1.37	0.62–3.00	0.4
Any cannabis use									
No				–	–	–	–	–	–
Yes				1.41	0.68–2.94	0.3	1.39	0.56–3.42	0.5
Any amphetamine use									
No				–	–	–	–	–	–
Yes				1.41	0.54–3.67	0.5	1.44	0.39–5.32	0.6
Any other drug use									
No				–	–	–	–	–	–
Yes				1.21	0.30–4.94	0.8	1.14	0.24–5.37	0.9
Peer substance use									
None				–	–	–	–	–	–
Some				1.41	0.53–3.79	0.5	1.08	0.45–2.58	0.9
Most				1.13	0.32–4.04	0.8	1.03	0.27–3.98	>0.9
Parental smoking									
Never/infrequent				–	–	–	–	–	–
Frequent/everyday				0.7	0.46–1.07	0.1	0.79	0.42–1.49	0.5
Parental alcohol use									
Never/infrequent				–	–	–	–	–	–
Frequent/everyday				1.05	0.55–2.00	0.9	0.95	0.47–1.91	0.9
Anxiety score at wave 6				1.41	1.06–1.89	0.02	1.31	0.94–1.81	0.11
Depression score at wave 6				1.6	1.21–2.10	0.001	1.4	1.01–1.96	0.05
Impulsivity score				0.94	0.87–1.01	0.1	0.94	0.86–1.04	0.2

Abbreviations: Aus, Australia; IPTW, inverse probability treatment weighting; MDMA, 3,4‐methylenedioxymethamphetamine.

## DISCUSSION

This study tested the relative longer‐term effects of different patterns of MDMA use across peak use in early adulthood (20–29 years) on meeting the criteria anxiety or depression diagnoses by the mid‐30s using prospective data collected in a large Australian, population based, longitudinal study of health and development. In early adulthood, 25% of the sample reported any MDMA use. Prevalence of past 12‐month use were highest at age ~24 at 18.5%. By the mid‐30s, 11% met the criteria for any anxiety diagnosis and 12% met the criteria for a major depressive disorder diagnosis.

Using statistical techniques to improve causal inference, specifically doubly robust IPTW methods, we found limited evidence of an association between MDMA use and later diagnosis of major depressive disorder. These findings are consistent with those of Daumann *et al*. [13] and George *et al*. [14], where after adjusting for prior experiences of mental health and other substance use, no evidence of a relationship was found between MDMA use and an increased likelihood of depression diagnosis. Further, in adolescents, Brière *et al*. [15] found that MDMA use no longer predicted depressive symptoms after controlling for other substance use, pre‐existing mental health, family and environmental factors.

We did, however, find that patterns of MDMA use in early adulthood raised the odds of meeting the criteria for past 12‐month anxiety disorder diagnosis by the mid‐30s. Likewise, the odds of an anxiety disorder diagnosis were double in participants who reported using MDMA at two or more waves in early adulthood compared to those who never used. This was also similar across frequency of use, where the odds of an anxiety diagnosis at 35 years were more than doubled in those who reported MDMA use at a frequency greater than monthly in the past 12‐months compared with no use. These findings were robust to further adjustment for other substance use, family factors, peer substance use, general health and prior mental health.

Pre‐clinical models have consistently found long term increases in anxiety‐like behaviour in behavioural tests among MDMA‐exposed rats compared to controls in the weeks to months following exposure [43, 44]. MDMA depletes neurotransmitter systems, mainly 5HT (serotonin), which is posited to increase anxiety‐like responses, possibly in a dose dependant manner. This disruption to normal functioning of neural circuits implicated in anxiety regulation can cause the brain to remain in a state of heightened arousal and vigilance, common to anxiety but not depression [45]. This may provide some explanation as to why there was no observed effect of MDMA on depression in the current study. The main findings in the current study are consistent with pre‐clinical literature. Neurotoxicity to 5HT containing neurons, which has been observed in pre‐clinical animal models such as rodents and non‐human primates, may be one mechanism behind these sustained changes. However, generalising results from animals to humans is complex and requires further investigation [4, 46]. Other indirect mechanisms may be potential persistent alterations in sleep induced by MDMA use. Differences in sleep between people who have previously used MDMA have been previously observed and are largely attributed to the extent of previous MDMA use [47].

### Strengths

The current study showcases several strengths that improve knowledge on the relationship between MDMA use and subsequent anxiety or depression diagnosis in midlife. The longitudinal nature of the data improves researchers understanding of a unidirectional temporal sequencing between exposure and outcome, allowing for improved causal estimates. The current study boasts a large sample size, which provides greater statistical power to improve the precision around effect estimates and greater power to detect effects. This study uses contemporary statistical approaches to enhance causal inference, specifically doubly robust IPTW methods that are more robust than traditional confounder adjustment techniques.

### Limitations

Frequency of MDMA use was not measured at wave 8 and past 12‐month any MDMA use increased from 7.6% at wave 7 to 18.5% at wave 8. Missing information in this peak use period may be impacting results for the frequency of MDMA use variable, such that the current figures may be an underestimation. The current study applied a simple causal model that did not consider the possibility of time‐varying confounding, when confounders have values that may change over time and could occur during the exposure period. Because of the scope and structure of substance use items, the influence of polysubstance on findings remains unclear. Polysubstance use includes the simultaneous or sequential use of multiple substances and has been found to be associated with greater risk of comorbid anxiety and depressive symptoms when compared to single substance use in isolation [48]. The current study controlled for other substance use, however, it was not possible to control for polysubstance use and may be another factor that may explain increase odds of anxiety diagnosis among the current sample. More research is needed to better understand whether polysubstance use confers long‐term risk for anxiety or depression diagnosis particularly among people who use MDMA, a highly prevalent polysubstance using group [2]. The current study did not measure quantity of MDMA use indicators so the degree in which participants are using per session is unclear and may impact associations. Additionally, while this study applied robust adjustment techniques using a rich set of measured confounders, and performed further sensitivity analyses, we recognise that unmeasured variables, such as early life adversity or trauma, may still influence both MDMA use and later mental health outcomes. We also acknowledge that 1992 Victorian adolescent cohort, who would have been ~21 to 22 years old (typical age of initiation to MDMA use) in 2000, may limit the generalisability of our findings to more contemporary populations.

Finally, the possibility of unmeasured confounding cannot be ruled out. We acknowledge that residual confounding because of unmeasured initiation or escalation of use between ages 18 and 20 cannot be fully ruled out. Further, recent research has highlighted that childhood adversity, early life trauma and psychological trauma are strong risk factors for both mental health and substance use [49, 50]. The current study did not measure such indicators of stressful life events until the final wave of data collection so they could not be considered confounders in this analysis [51]. Although the current study controlled for proxy variables in lieu of these measures, trauma may play a role in the relationship between MDMA use and anxiety. Future studies should consider the impact of childhood adversity/early life trauma indicators on the relationship between MDMA use and anxiety diagnosis.

### Public health and clinical implications

Harm minimisation campaigns that aim to increase awareness of the potential mental health risks of recreational MDMA use should be considered as interventions, particularly among high‐risk populations such as those who use more frequently or persistently. From a prevention perspective it is also important to consider evidence‐based programs in the high school setting targeting MDMA use and related substances, such as the Our Futures: MDMA and emerging drugs module (https://ourfuturesinstitute.org.au/) before typical onset of use.

Over the past decade, research into the therapeutic use of MDMA‐assisted psychotherapy has increased exponentially, however, evidence of effectiveness is still in its infancy. Although some clinical trials involving MDMA have reported adverse events, including increased odds of anxiety at follow‐up (e.g. 7 days post‐treatment), it is difficult to equate these findings with those from recreational MDMA use, as examined in the current study [52]. This likely reflects key differences in dosing (clinical trials typically involve 1–3 controlled sessions), as well as in drug purity, production conditions and the presence of known or unknown adulterants in recreational settings [53, 54]. Nevertheless, this study highlights the need for greater population and clinical evidence base on both recreational and clinical use of MDMA, emphasising the need for long term follow‐ups of clinical trial participants to better understand whether these findings deviate from the clinical setting.

## CONCLUSIONS

This study examined the long‐term effects of MDMA use on anxiety or depression diagnosis in midlife. Although no significant association was found between MDMA use and depression diagnosis, a notable link was observed between MDMA use and increased odds of any past 12‐month anxiety disorder diagnosis in midlife. Although the current study positions researchers closer to understanding the potential causal associations between MDMA use, anxiety and depression, a greater understanding of polysubstance use and experiences of trauma among people who use MDMA would further improve confidence in findings and should be considered in future research.

## AUTHOR CONTRIBUTIONS

**Zachary Bryant:** Conceptualization (lead); formal analysis (lead); investigation (lead); methodology (lead); project administration (lead); visualization (lead); writing—original draft (lead); writing—review and editing (lead). **Kirsten Morley:** Conceptualization (supporting); formal analysis (supporting); investigation (supporting); project administration (supporting); supervision (equal); writing—review and editing (supporting). **Jessica A. Kerr:** Data curation (equal); project administration (supporting); writing—review and editing (supporting). **Craig A. Olsson:** Data curation (equal); writing—review and editing (supporting). **Tim Slade:** Conceptualization (supporting); formal analysis (supporting); investigation (supporting); methodology (supporting); project administration (supporting); supervision (equal); writing—original draft (supporting); writing—review and editing (supporting).

## DECLARATION OF INTERESTS

None.

## Supporting information

Supporting Information.

## Data Availability

The data that support the findings of this study are available from the Murdoch Childrens Research Institute. Restrictions apply to the availability of these data, which were used under license for this study. For more information on data availability please visit https://www.mcri.edu.au/research/projects/2000-stories.
