# Supplementary material for: The relationship between 3,4‐methylenedioxymethamphetamine (MDMA) use in young adulthood and anxiety or depressive disorders in the mid‐30s: Findings from the Victorian Adolescent Health Cohort Study
Source: Addiction. 2025 Aug 28;120(12):2448–64. doi: 10.1111/add.70173 (PMC12586782; doi:10.1111/add.70173)
Supplement: Supplementary file 1 — Supporting Information. [file ADD-120-2448-s001.docx]

**Supplementary materials**

| **Table S1. Descriptive participant characteristics of exposure, outcome and confounding variables** | | | | | | | | | |
| --- | --- | --- | --- | --- | --- | --- | --- | --- | --- |
|  | **Any MDMA use**  (N = 1329) | | **Any persistent MDMA use**  (N = 1329) | | | **Any frequency of MDMA use***  (N = 1221) | | | **Total sample** (N = 1329) |
| **Characteristic** | **Never used**  (N = 994) | **Any use**  (N = 335) | **Use at no waves**  (N = 994) | **Use at 1 wave** (N = 201) | **Use at 2 or more waves**  (N = 134) | **Never used frequently**  (N = 994) | **Infrequent or experimental use**  (N = 166) | **Frequent use** (N = 61) |  |
| ***Sex*** |  |  |  |  |  |  |  |  |  |
| Female | 577 (58%) | 150 (45%) | 577 (58%) | 94 (47%) | 56 (42%) | 577 (58%) | 72 (43%) | 26 (43%) | 727 (55%) |
| Male | 417 (42%) | 185 (55%) | 417 (42%) | 107 (53%) | 78 (58%) | 417 (42%) | 94 (57%) | 35 (57%) | 602 (45%) |
| Missing | 0 (0%) | 0 (0%) | 0 (0%) | 0 (0%) | 0 (0%) | 0 (0%) | 0 (0%) | 0 (0%) | 0 (0%) |
| ***Nationality*** |  |  |  |  |  |  |  |  |  |
| Born in Australia | 833 (84%) | 303 (90%) | 833 (84%) | 182 (91%) | 121 (90%) | 833 (84%) | 149 (90%) | 53 (87%) | 1,136 (85%) |
| Born outside of Australia | 125 (13%) | 27 (8%) | 125 (13%) | 15 (7%) | 12 (9%) | 125 (13%) | 13 (8%) | 7 (11%) | 152 (11%) |
| Missing | 36 (4%) | 5 (1%) | 36 (4%) | 4 (2%) | 1 (1%) | 36 (4%) | 4 (2%) | 1 (2%) | 41 (4%) |
| ***Index of Relative Socio-economic Disadvantage (1995)*** |  |  |  |  |  |  |  |  |  |
| Quintile 1 (most disadvantaged) | 127 (13%) | 26 (8%) | 127 (13%) | 16 (8%) | 10 (7%) | 127 (13%) | 11 (7%) | 4 (7%) | 153 (12%) |
| Quintile 2 | 140 (14%) | 31 (9%) | 140 (14%) | 19 (9%) | 12 (9%) | 140 (14%) | 13 (8%) | 6 (10%) | 171 (13%) |
| Quintile 3 | 125 (13%) | 40 (12%) | 125 (13%) | 21 (10%) | 19 (14%) | 125 (13%) | 22 (13%) | 6 (10%) | 165 (12%) |
| Quintile 4 | 218 (22%) | 68 (20%) | 218 (22%) | 45 (22%) | 23 (17%) | 218 (22%) | 33 (20%) | 14 (23%) | 286 (22%) |
| Quintile 5 (least disadvantaged) | 372 (37%) | 167 (50%) | 372 (37%) | 98 (49%) | 69 (51%) | 372 (37%) | 87 (52%) | 30 (49%) | 539 (40%) |
| Missing | 12 (1%) | 3 (1%) | 12 (1%) | 2 (<1%) | 1 (1%) | 12 (1%) | 0 (0%) | 1 (2%) | 15 (1%) |
| ***Parental divorce or separation*** |  |  |  |  |  |  |  |  |  |
| No | 818 (83%) | 263 (79%) | 818 (83%) | 152 (76%) | 111 (83%) | 818 (83%) | 135 (81%) | 48 (79%) | 1081 (81%) |
| Yes | 173 (17%) | 71 (21%) | 173 (17%) | 48 (24%) | 23 (17%) | 173 (17%) | 30 (18%) | 13 (21%) | 244 (18%) |
| Missing | 3 (<1%) | 1 (<1%) | 3 (<1%) | 1 (<1%) | 0 (0%) | 3 (<1%) | 1 (1%) | 0 (0%) | 4 (1%) |
| ***General health*** |  |  |  |  |  |  |  |  |  |
| Excellent | 204 (21%) | 63 (19%) | 204 (21%) | 46 (23%) | 17 (13%) | 204 (21%) | 29 (17%) | 12 (20%) | 267 (20%) |
| Good | 467 (47%) | 156 (47%) | 467 (47%) | 88 (44%) | 68 (51%) | 467 (47%) | 83 (50%) | 29 (48%) | 623 (47%) |
| Fair | 157 (16%) | 64 (19%) | 157 (16%) | 37 (18%) | 27 (20%) | 157 (16%) | 29 (17%) | 11 (18%) | 221 (17%) |
| Poor | 18 (2%) | 8 (2%) | 18 (2%) | 4 (2%) | 4 (3%) | 18 (2%) | 4 (2%) | 2 (3%) | 26 (2%) |
| Missing | 148 (15%) | 44 (13%) | 148 (15%) | 26 (13%) | 18 (13%) | 148 (15%) | 21 (13%) | 7 (11%) | 192 (14%) |
| ***Any past 12-month smoking*** |  |  |  |  |  |  |  |  |  |
| No | 659 (66%) | 153 (46%) | 659 (66%) | 106 (53%) | 47 (35%) | 659 (66%) | 73 (44%) | 26 (43%) | 812 (61%) |
| Yes | 193 (20%) | 142 (42%) | 193 (20%) | 72 (36%) | 70 (52%) | 193 (20%) | 75 (45%) | 29 (47%) | 335 (25%) |
| Missing | 142 (14%) | 40 (12%) | 142 (14%) | 23 (11%) | 17 (13%) | 142 (14%) | 18 (11%) | 6 (10%) | 182 (14%) |
| ***Any past 12-month high-risk alcohol use*** |  |  |  |  |  |  |  |  |  |
| No | 727 (73%) | 186 (55%) | 727 (73%) | 121 (60%) | 65 (48%) | 727 (73%) | 95 (57%) | 33 (54%) | 913 (67%) |
| Yes | 125 (13%) | 109 (33%) | 125 (13%) | 57 (28%) | 52 (39%) | 125 (13%) | 53 (32%) | 22 (36%) | 234 (18%) |
| Missing | 142 (14%) | 40 (12%) | 142 (14%) | 23 (11%) | 17 (13%) | 142 (14%) | 18 (11%) | 6 (10%) | 182 (15%) |
| ***Any past 12-month cannabis use*** |  |  |  |  |  |  |  |  |  |
| No | 747 (75%) | 146 (43%) | 747 (75%) | 102 (51%) | 44 (33%) | 747 (75%) | 74 (45%) | 20 (33%) | 893 (67%) |
| Yes | 95 (10%) | 143 (43% | 95 (10%) | 71 (35%) | 72 (54%) | 95 (10%) | 70 (42%) | 33 (54%) | 238 (18%) |
| Missing | 152 (15%) | 46 (14%) | 152 (15%) | 28 (14%) | 18 (13%) | 152 (15%) | 22 (13%) | 8 (13%) | 198 (15%) |
| ***Any past 12-month amphetamine use*** |  |  |  |  |  |  |  |  |  |
| No | 822 (83%) | 257 (77%) | 822 (83%) | 155 (77%) | 102 (76%) | 822 (83%) | 130 (78%) | 46 (76%) | 1079 (81%) |
| Yes | 5 (<1%) | 26 (8%) | 5 (<1%) | 15 (7%) | 11 (8%) | 5 (<1%) | 11 (7%) | 7 (11%) | 31 (2%) |
| Missing | 167 (17%) | 52 (15%) | 167 (17%) | 31 (15%) | 21 (16%) | 167 (17%) | 25 (15%) | 8 (13%) | 219 (17%) |
| ***Any past 12-month other drug use*** |  |  |  |  |  |  |  |  |  |
| No | 813 (82%) | 245 (73%) | 813 (82%) | 148 (74%) | 97 (73%) | 813 (82%) | 121 (73%) | 45 (74%) | 1058 (80%) |
| Yes | 15 (1%) | 30 (9%) | 15 (1%) | 16 (8%) | 14 (10%) | 15 (1%) | 16 (10%) | 7 (11%) | 45 (3%) |
| Missing | 166 (17%) | 60 (18%) | 166 (17%) | 37 (18%) | 23 (17%) | 166 (17%) | 29 (17%) | 9 (15%) | 226 (17%) |
| ***Peer alcohol use*** |  |  |  |  |  |  |  |  |  |
| None | 344 (35%) | 45 (13%) | 344 (35%) | 34 (17%) | 11 (8%) | 344 (35%) | 26 (16%) | 5 (8%) | 389 (29%) |
| Some | 367 (37%) | 165 (49%) | 367 (37%) | 94 (47%) | 71 (53%) | 367 (37%) | 88 (53%) | 29 (48%) | 532 (40%) |
| Most | 41 (4%) | 69 (21%) | 41 (4%) | 39 (19%) | 30 (22%) | 41 (4%) | 26 (16%) | 18 (30%) | 110 (8%) |
| Missing | 242 (24%) | 56 (17%) | 242 (24%) | 34 (17%) | 22 (16%) | 242 (24%) | 26 (16%) | 9 (15%) | 298 (23%) |
| ***Parental smoking*** |  |  |  |  |  |  |  |  |  |
| Never/Infrequent | 601 (60%) | 196 (59%) | 601 (60%) | 116 (58%) | 80 (60%) | 601 (60%) | 103 (62%) | 32 (52%) | 797 (60%) |
| Frequent/everyday | 234 (24%) | 91 (27%) | 234 (24%) | 57 (28%) | 34 (25%) | 234 (24%) | 40 (24%) | 21 (34%) | 325 (24%) |
| Missing | 159 (16%) | 48 (14%) | 159 (16%) | 28 (14%) | 20 (15%) | 159 (16%) | 23 (14%) | 8 (13%) | 207 (16%) |
| ***Parental alcohol use*** |  |  |  |  |  |  |  |  |  |
| Never/Infrequent | 641 (64%) | 181 (54%) | 641 (64%) | 107 (53%) | 74 (55%) | 641 (64%) | 90 (54%) | 34 (56%) | 822 (62%) |
| Frequent/everyday | 207 (21%) | 113 (34%) | 207 (21%) | 70 (35%) | 43 (32%) | 207 (21%) | 57 (34%) | 21 (34%) | 320 (24%) |
| Missing | 146 (15%) | 41 (12%) | 146 (15%) | 24 (12%) | 17 (13%) | 146 (15%) | 19 (11%) | 6 (10%) | 187 (14%) |
| ***Anxiety score at wave 6 (mean/SD)*** | 0.2 (0.6) | 0.3 (0.8) | 0.2 (0.6) | 0.3 (0.8) | 0.3 (0.7) | 0.2 (0.6) | 0.4 (0.9) | 0.1 (0.5) | 0.2 (0.7) |
| Missing | 142 (14%) | 40 (12%) | 142 (14%) | 23 (11%) | 17 (13%) | 142 (14%) | 18 (11%) | 6 (10%) | 182 (14%) |
| ***Depression score at wave 6 (mean/SD)*** | 0.3 (0.7) | 0.3 (0.8) | 0.3 (0.7) | 0.7 (0.7) | 0.4 (0.9) | 0.3 (0.7) | 0.4 (0.9) | 0.3 (0.8) | 0.3 (0.7) |
| Missing | 142 (14%) | 40 (12%) | 142 (14%) | 23 (11%) | 17 (13%) | 142 (14%) | 18 (11%) | 6 (10%) | 182 (14%) |
| ***Impulsivity score at wave 2 (mean/SD)*** | 4.6 (3) | 5.6 (3.2) | 4.6 (3) | 5.1 (3) | 6.3 (3.4) | 4.6 (3) | 5.8 (3.4) | 5.8 (3.1) | 4.9 (3.1) |
| Missing | 54 (5%) | 21 (6%) | 54 (5%) | 13 (6%) | 8 (6%) | 54 (5%) | 11 (7%) | 6 (10%) | 75 (6%) |
| ***Any anxiety disorder diagnosis wave 10*** |  |  |  |  |  |  |  |  |  |
| No | 898 (90%) | 280 (84%) | 898 (90%) | 168 (84%) | 112 (84%) | 898 (90%) | 137 (82%) | 48 (79%) | 1178 (89%) |
| Yes | 96 (10%) | 55 (16%) | 96 (10%) | 33 (16%) | 22 (16%) | 96 (10%) | 29 (18%) | 13 (21%) | 151 (11%) |
| Missing | 0 (0%) | 0 (0%) | 0 (0%) | 0 (0%) | 0 (0%) | 0 (0%) | 0 (0%) | 0 (0%) | 0 (0%) |
| ***Depression diagnosis wave 10*** |  |  |  |  |  |  |  |  |  |
| No | 887 (89%) | 289 (86%) | 887 (89%) | 176 (88%) | 113 (84%) | 887 (89%) | 143 (86%) | 51 (84%) | 1176 (88%) |
| Yes | 107 (11%) | 46 (14%) | 107 (11%) | 25 (12%) | 21 (16%) | 107 (11%) | 23 (14%) | 10 (16%) | 153 (12%) |
| Missing | 0 (0%) | 0 (0%) | 0 (0%) | 0 (0%) | 0 (0%) | 0 (0%) | 0 (0%) | 0 (0%) | 0 (0%) |

* Reduced sample size due to wave 8 frequency of use data not available – 108 cases of ‘any use’ only reported at wave 8 were removed.

**Effect of weight truncation on IPT weights**

The IPTW generates unstabilised weights which can result in extreme values i.e. if an individual is highly unlikely to be in an exposure group based on the observed covariates but is nonetheless in the exposure group, the analyses will be heavily influenced by those individuals (1). This often increases the variance, and subsequent uncertainty of effect estimates, therefore we tested the effect of varying degrees of weight truncation on covariate balance between exposure groups and effect estimates. After creating the initial IPT weights for each participant, extreme cases were observed. To handle extreme weights, we assessed changes in mean weight, variance, and effect estimate under 4 weight truncation conditions at the unadjusted, 99^th^, 95^th^ and 90^th^ percentiles.

As seen below in tables S1-7 truncating at the 99^th^ percentile reduces the variance across the models, however the maximum weights may still be considered extreme values. When truncating at the 95^th^ percentile the covariate balance across exposure groups is still stable and within a maximum standardised mean difference between groups of ~0.1 as seen below in figures S1-12 below (2). When weights are truncated at the 90^th^ percentile, the spread of covariates across exposure groups become unbalanced. Therefore, it was decided that truncating all models at the 95^th^ percentile provides the best balance between reducing the influence of extreme weights, maintaining covariate balance across exposure groups and improving precision in effect estimates.

| **Table S2. Weight truncation for any MDMA use x depression** | | | | | |
| --- | --- | --- | --- | --- | --- |
|  | **Mean (sd)** | **Min - max** | **OR** | **CI** | **p-value** |
| **Unadjusted weights** | 2 (2) | 1-20.1 | **-** | **-** | **-** |
| Any MDMA use - yes | **-** | **-** | 0.99 | 0.63, 1.56 | >0.9 |
| **Trunc weights (99^th^%)** | 2 (1.9) | 1-12.2 | **-** | **-** | **-** |
| Any MDMA use - yes | **-** | **-** | 1.13 | 0.72, 1.78 | 0.6 |
| **Trunc weights (95^th^%)** | 1.9 (1.4) | 1-6.5 | **-** | **-** | **-** |
| Any MDMA use - yes | **-** | **-** | 1.12 | 0.73, 1.73 | 0.6 |
| **Trunc weights (90^th^%)** | 1.7 (0.9) | 1-4.1 | **-** | **-** | **-** |
| Any MDMA use - yes | **-** | **-** | 1.16 | 0.76, 1.76 | 0.5 |

| **Table S3: Weight truncation for any frequent MDMA use x depression** | | | | | |
| --- | --- | --- | --- | --- | --- |
|  | **Mean (sd)** | **Min - max** | **OR** | **CI** | **p-value** |
| **Unadjusted weights** | 3.2 (7.6) | 1-155.8 | **-** | **-** | **-** |
| Infrequent/experimental | **-** | **-** | 0.95 | 0.50, 1.81 | 0.9 |
| Frequent | **-** | **-** | 1.67 | 0.61, 4.58 | 0.3 |
| **Trunc weights (99^th^%)** | 3 (5.5) | 1-43.5 | **-** | **-** | **-** |
| Infrequent/experimental | **-** | **-** | 0.94 | 0.51, 1.76 | 0.9 |
| Frequent | **-** | **-** | 1.62 | 0.62, 4.27 | 0.3 |
| **Trunc weights (95^th^%)** | 2.4 (2.9) | 1-13.1 | **-** | **-** | **-** |
| Infrequent/experimental | **-** | **-** | 1.04 | 0.59, 1.84 | 0.9 |
| Frequent | **-** | **-** | 1.5 | 0.62, 3.63 | 0.4 |
| **Trunc weights (90^th^%)** | 2 (1.7) | 1-7.1 | **-** | **-** | **-** |
| Infrequent/experimental | **-** | **-** | 1.13 | 0.66, 1.94 | 0.6 |
| Frequent | **-** | **-** | 1.56 | 0.69, 3.52 | 0.3 |

| **Table S4: Weight truncation for any persistent MDMA use x depression** | | | | | |
| --- | --- | --- | --- | --- | --- |
|  | **Mean (sd)** | **Min - max** | **OR** | **CI** | **p-value** |
| **Unadjusted weights** | 3.1 (5.6) | 1-117.5 | **-** | **-** | **-** |
| Infrequent/experimental | **-** | **-** | 0.87 | 0.51, 1.49 | 0.6 |
| Frequent | **-** | **-** | 1.25 | 0.57, 2.77 | 0.6 |
| **Trunc weights (99^th^%)** | 2.9 (4.2) | 1-30.9 | **-** | **-** | **-** |
| Infrequent/experimental | **-** | **-** | 0.94 | 0.53, 1.67 | 0.8 |
| Frequent | **-** | **-** | 1.44 | 0.73, 2.82 | 0.3 |
| **Trunc weights (95^th^%)** | 2.6 (2.7) | 1-11.6 | **-** | **-** | **-** |
| Infrequent/experimental | **-** | **-** | 0.95 | 0.56, 1.61 | 0.8 |
| Frequent | **-** | **-** | 1.38 | 0.74, 2.60 | 0.3 |
| **Trunc weights (90^th^%)** | 2.3 (1.9) | 1-7.5 | **-** | **-** | **-** |
| Infrequent/experimental | **-** | **-** | 0.98 | 0.59, 1.64 | >0.9 |
| Frequent | **-** | **-** | 1.39 | 0.76, 2.54 | 0.3 |

| **Table S5. Weight truncation for any MDMA use x anxiety** | | | | | |
| --- | --- | --- | --- | --- | --- |
|  | **Mean (sd)** | **Min - max** | **OR** | **CI** | **p-value** |
| **Unadjusted weights** | 2 (2) | 1-20.1 | **-** | **-** | **-** |
| Any MDMA use - yes | **-** | **-** | 1.49 | 0.94, 2.37 | 0.089 |
| **Trunc weights (99^th^%)** | 2 (1.9) | 1-12.2 | **-** | **-** | **-** |
| Any MDMA use - yes | **-** | **-** | 1.71 | 1.09, 2.68 | 0.02 |
| **Trunc weights (95^th^%)** | 1.9 (1.4) | 1-6.5 | **-** | **-** | **-** |
| Any MDMA use - yes | **-** | **-** | 1.73 | 1.12, 2.68 | 0.014 |
| **Trunc weights (90^th^%)** | 1.7 (0.9) | 1-4.1 | **-** | **-** | **-** |
| Any MDMA use - yes | **-** | **-** | 1.68 | 1.15,2.46 | 0.008 |

| **Table S6: Weight truncation for any frequent MDMA use x anxiety** | | | | | |
| --- | --- | --- | --- | --- | --- |
|  | **Mean (sd)** | **Min - max** | **OR** | **CI** | **p-value** |
| **Unadjusted weights** | 3.2 (7.6) | 1-155.8 | **-** | **-** | **-** |
| Infrequent/experimental | **-** | **-** | 2.3 | 1.14, 4.61 | 0.019 |
| Frequent | **-** | **-** | 2.22 | 0.81, 6.07 | 0.12 |
| **Trunc weights (99^th^%)** | 3 (5.5) | 1-43.5 | **-** | **-** | **-** |
| Infrequent/experimental | **-** | **-** | 2.24 | 1.13, 4.43 | 0.02 |
| Frequent | **-** | **-** | 2.4 | 0.95, 6.09 | 0.065 |
| **Trunc weights (95^th^%)** | 2.4 (2.9) | 1-13.1 | **-** | **-** | **-** |
| Infrequent/experimental | **-** | **-** | 2.11 | 1.14, 3.92 | 0.018 |
| Frequent | **-** | **-** | 2.56 | 1.15, 5.71 | 0.022 |
| **Trunc weights (90^th^%)** | 2 (1.7) | 1-7.1 | **-** | **-** | **-** |
| Infrequent/experimental | **-** | **-** | 2.1 | 1.19, 3.72 | 0.011 |
| Frequent | **-** | **-** | 2.6 | 1.21, 5.62 | 0.015 |

| **Table S7: Weight truncation for any persistent MDMA use x anxiety** | | | | | |
| --- | --- | --- | --- | --- | --- |
|  | **Mean (sd)** | **Min - max** | **OR** | **CI** | **p-value** |
| **Unadjusted weights** | 3.1 (5.6) | 1-117.5 | **-** | **-** | **-** |
| Infrequent/experimental | **-** | **-** | 1.29 | 0.73, 2.30 | 0.4 |
| Frequent | **-** | **-** | 2.31 | 1.05, 5.05 | 0.036 |
| **Trunc weights (99^th^%)** | 2.9 (4.2) | 1-30.9 | **-** | **-** | **-** |
| Infrequent/experimental | **-** | **-** | 1.61 | 0.94, 2.76 | 0.081 |
| Frequent | **-** | **-** | 2.1 | 1.02, 4.34 | 0.044 |
| **Trunc weights (95^th^%)** | 2.6 (2.7) | 1-11.6 | **-** | **-** | **-** |
| Infrequent/experimental | **-** | **-** | 1.6 | 0.96, 2.67 | 0.071 |
| Frequent | **-** | **-** | 2.05 | 1.07, 3.94 | 0.031 |
| **Trunc weights (90^th^%)** | 2.3 (1.9) | 1-7.5 | **-** | **-** | **-** |
| Infrequent/experimental | **-** | **-** | 1.61 | 0.99, 2.63 | 0.054 |
| Frequent | **-** | **-** | 1.99 | 1.06, 3.74 | 0.031 |

**Figure S1: Love plot displaying covariate balance for any MDMA exposure without weight truncation**

**
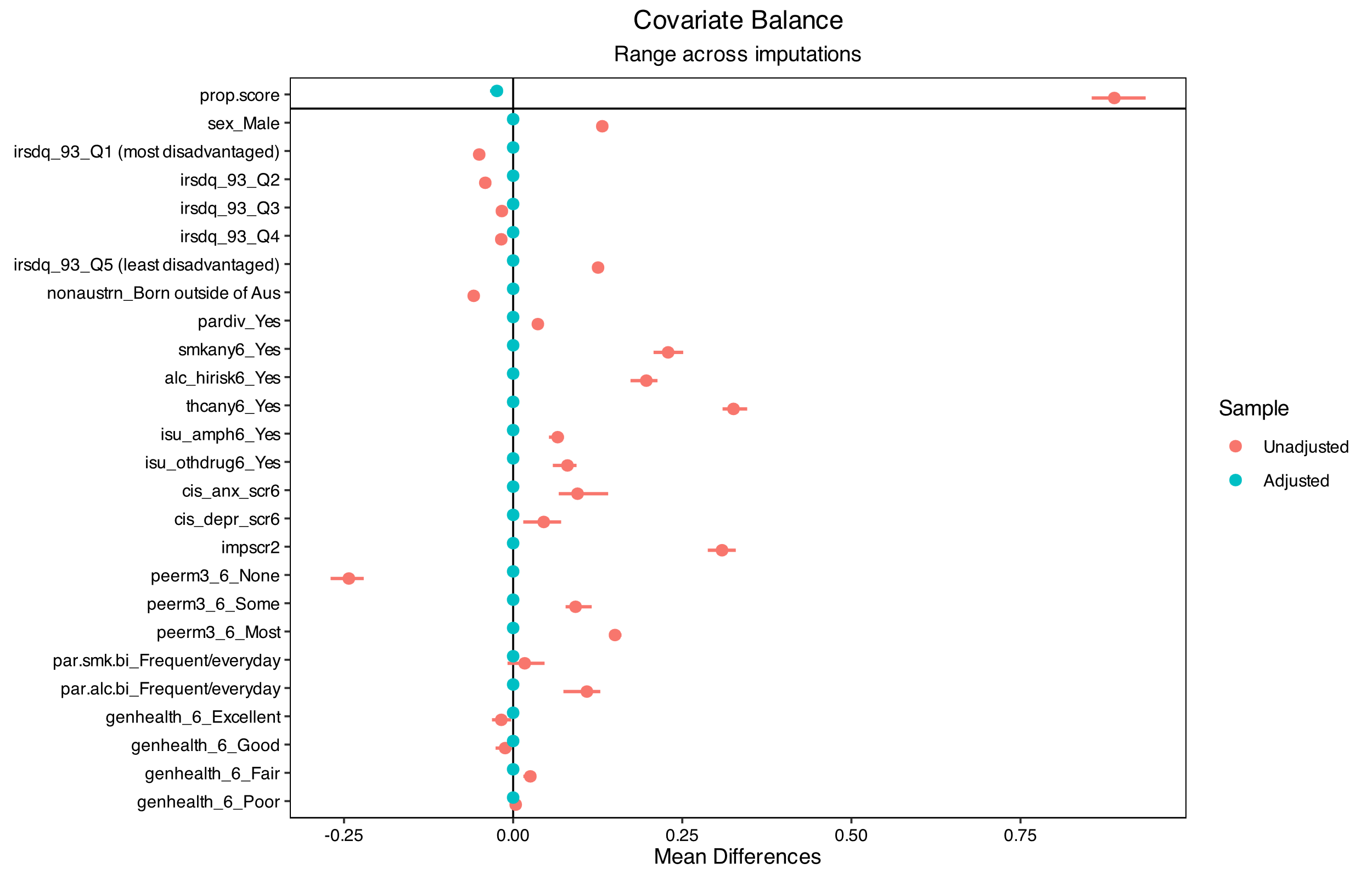
**

**Figure S2: Love plot displaying covariate balance for any MDMA exposure with weights truncated at 99^th^ percentile**

**
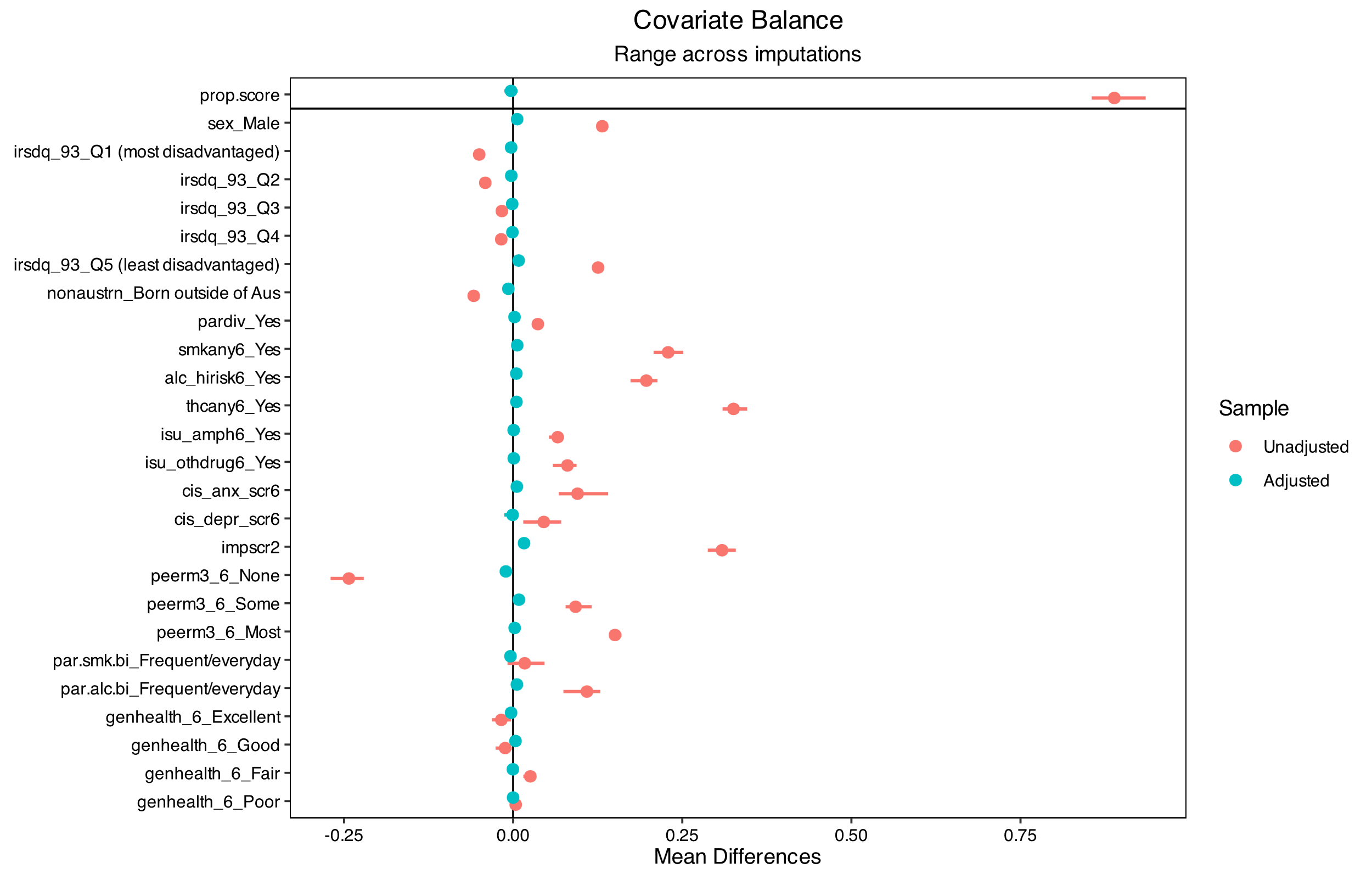
**

**Figure S3: Love plot displaying covariate balance for any MDMA exposure with weights truncated at 95^th^ percentile**

**
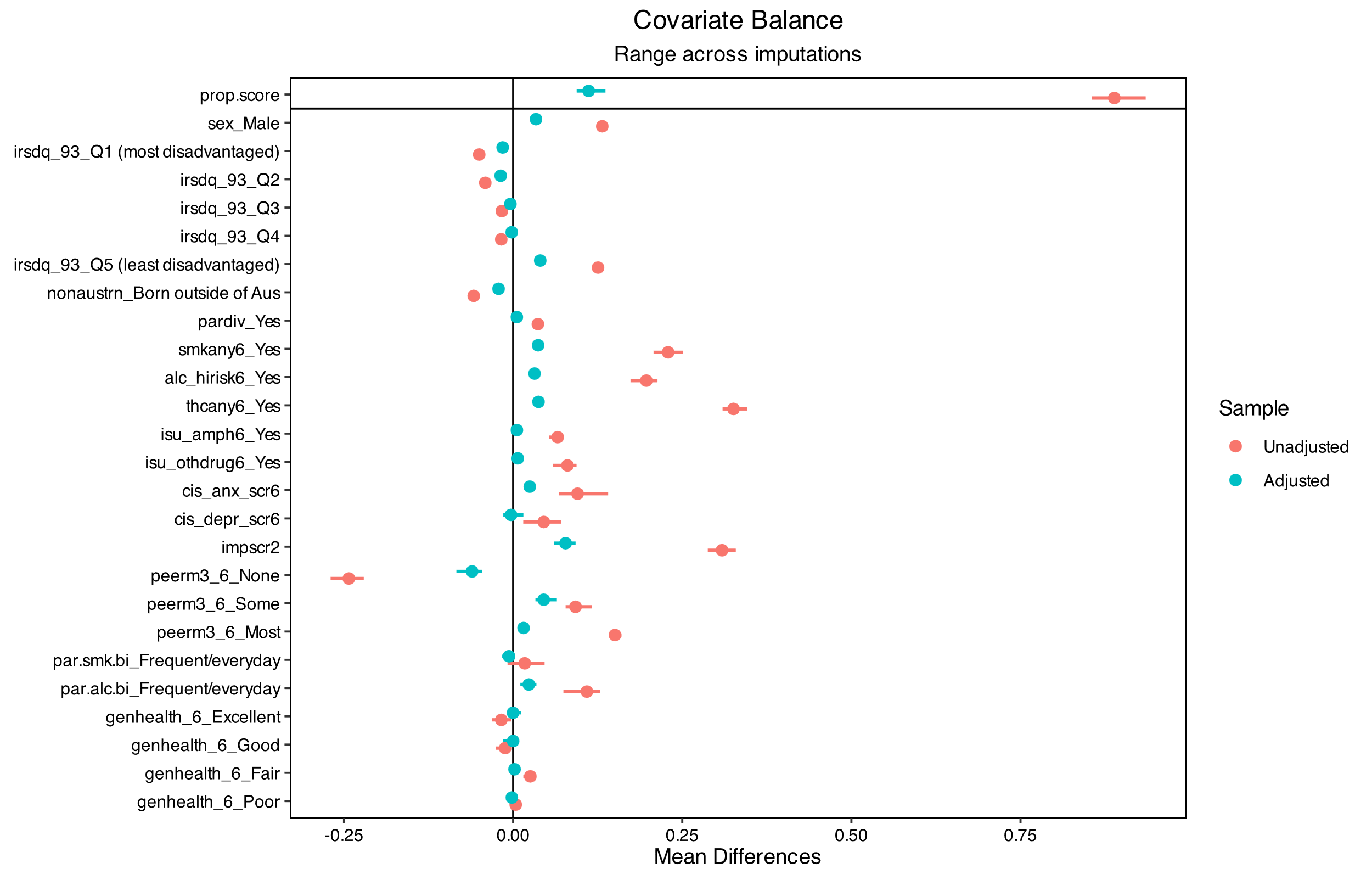
**

**Figure S4: Love plot displaying covariate balance for any MDMA exposure with weights truncated at 90^th^ percentile**

**
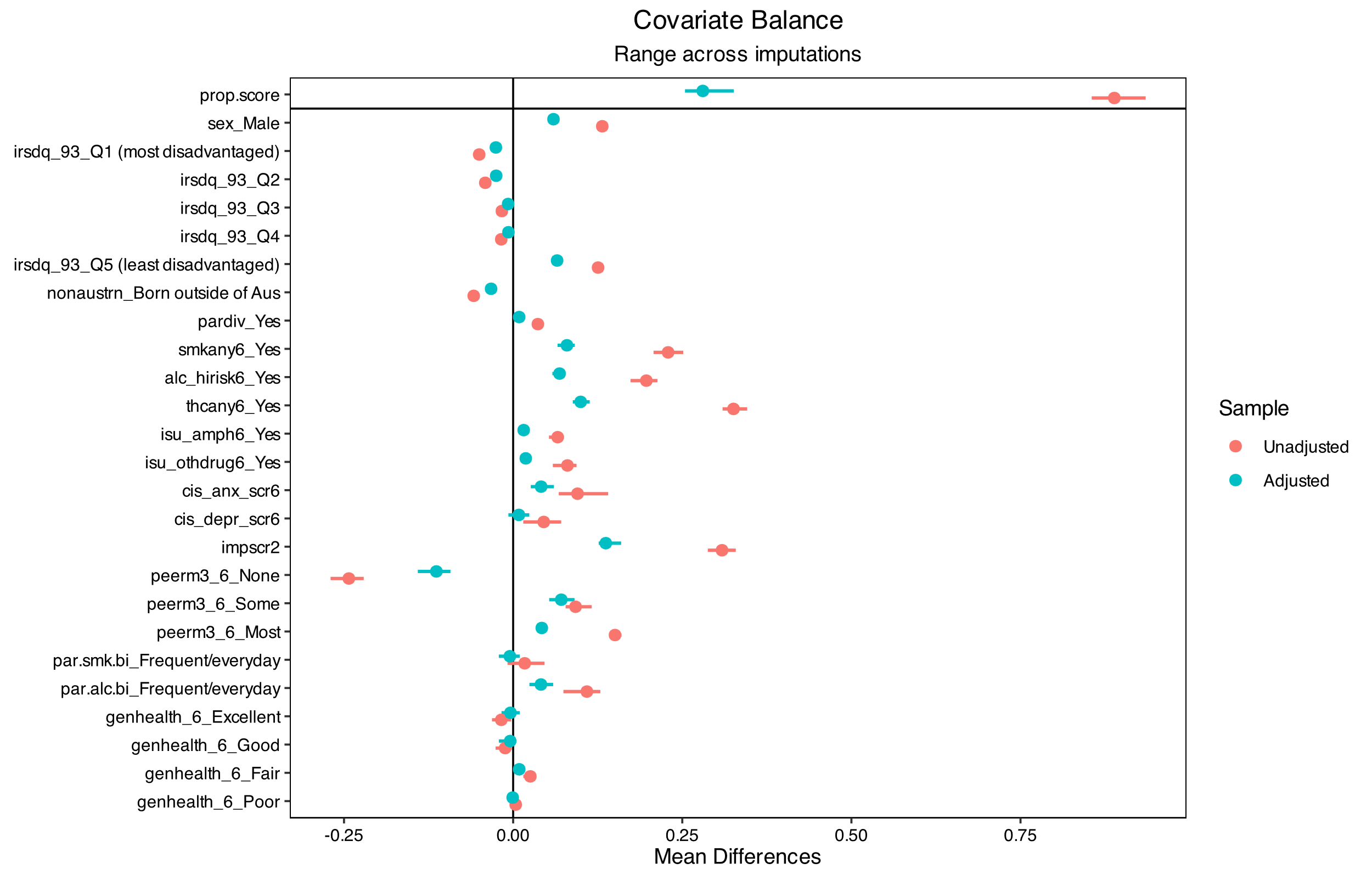
**

**Figure S5: Love plot displaying covariate balance for any frequent MDMA use exposure without weight truncation**


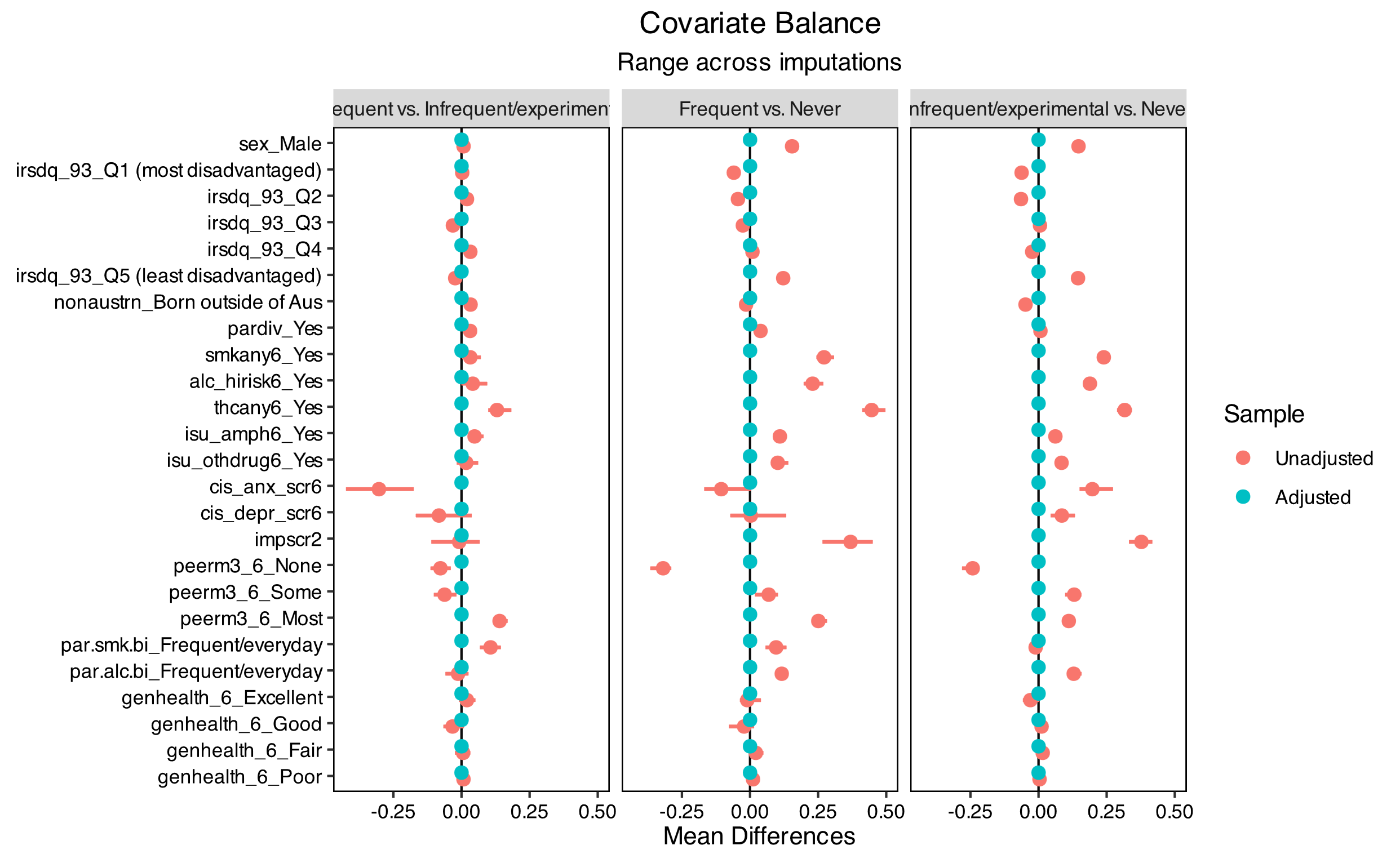


**Figure S6: Love plot displaying covariate balance for any frequent MDMA use exposure with weights truncated at 99^th^ percentile**


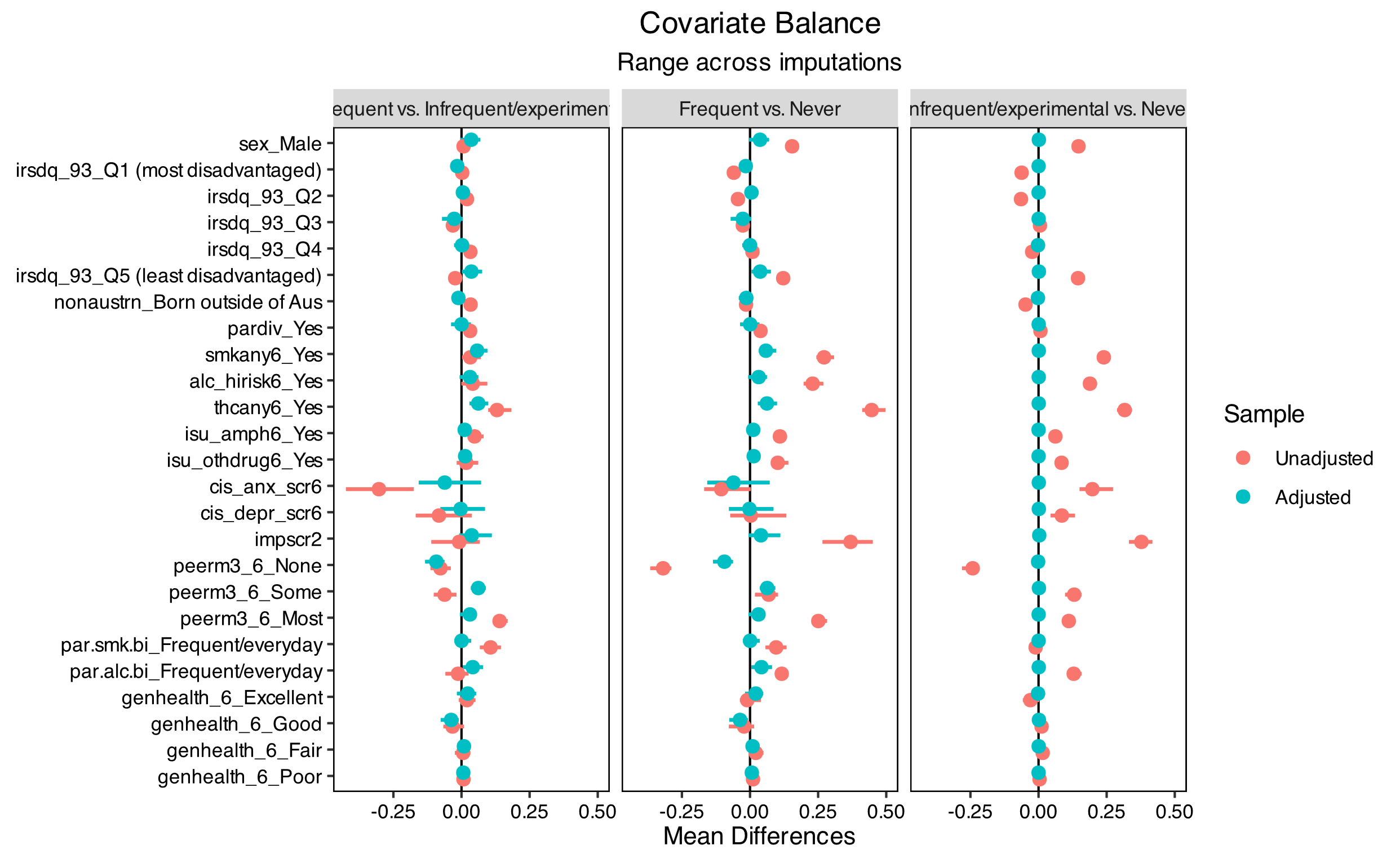


**Figure S7: Love plot displaying covariate balance for any frequent MDMA use exposure with weights truncated at 95^th^ percentile**


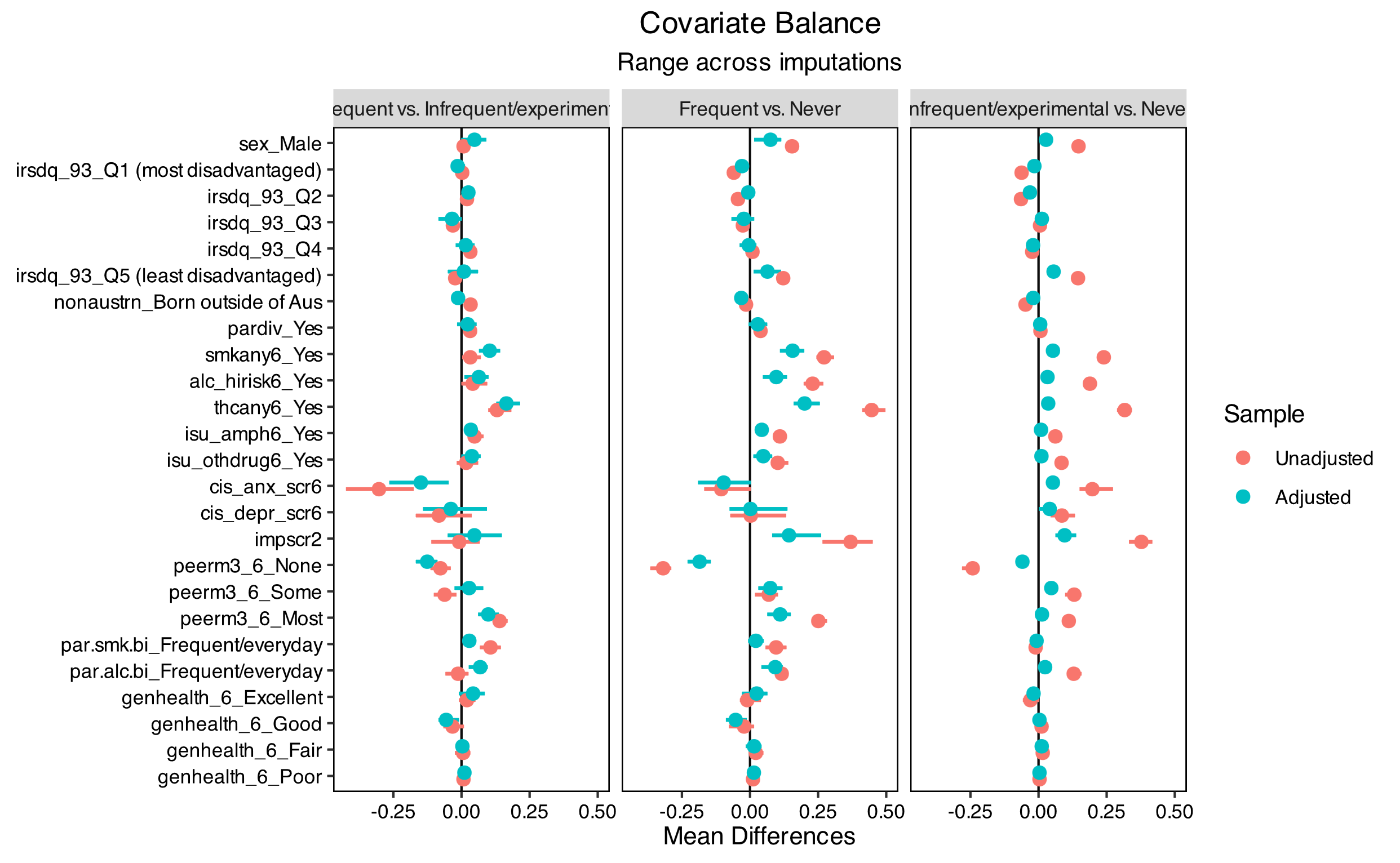


**Figure S8: Love plot displaying covariate balance for any frequent MDMA use exposure with weights truncated at 90^th^ percentile**


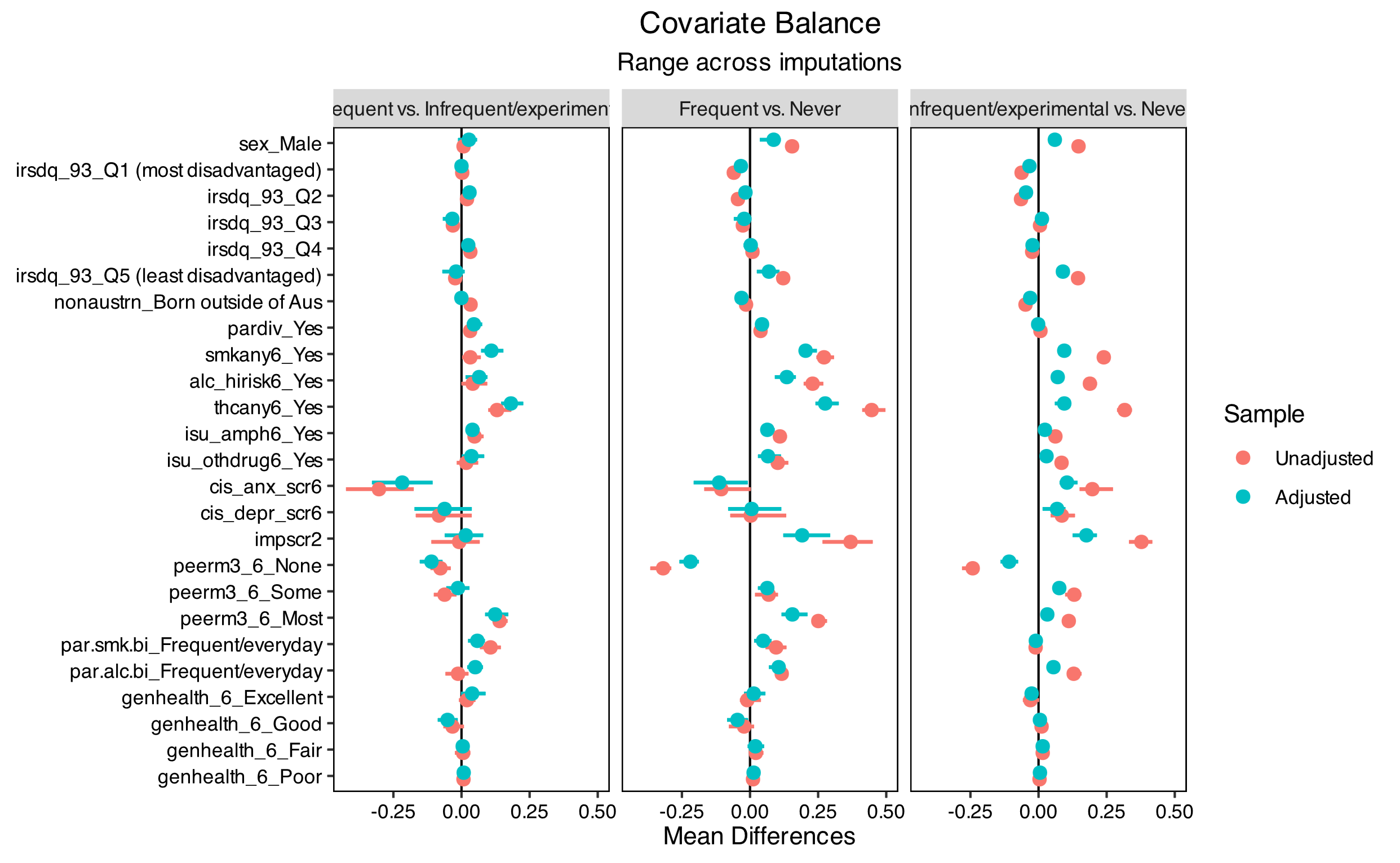


**Figure S9: Love plot displaying covariate balance for any persistent MDMA use exposure without weight truncation**


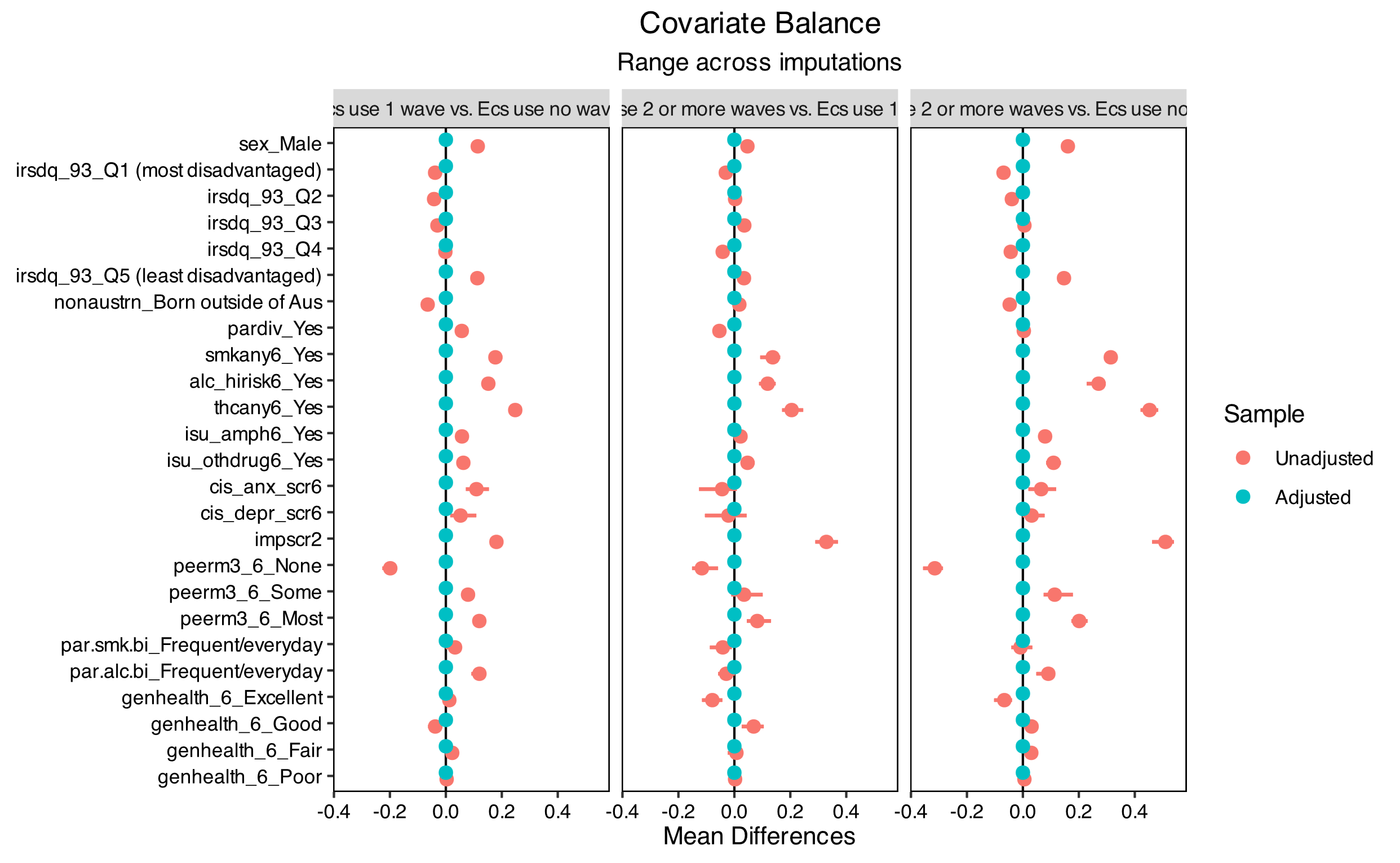


**Figure S10: Love plot displaying covariate balance for any persistent MDMA use exposure with weights truncated at 99^th^ percentile**


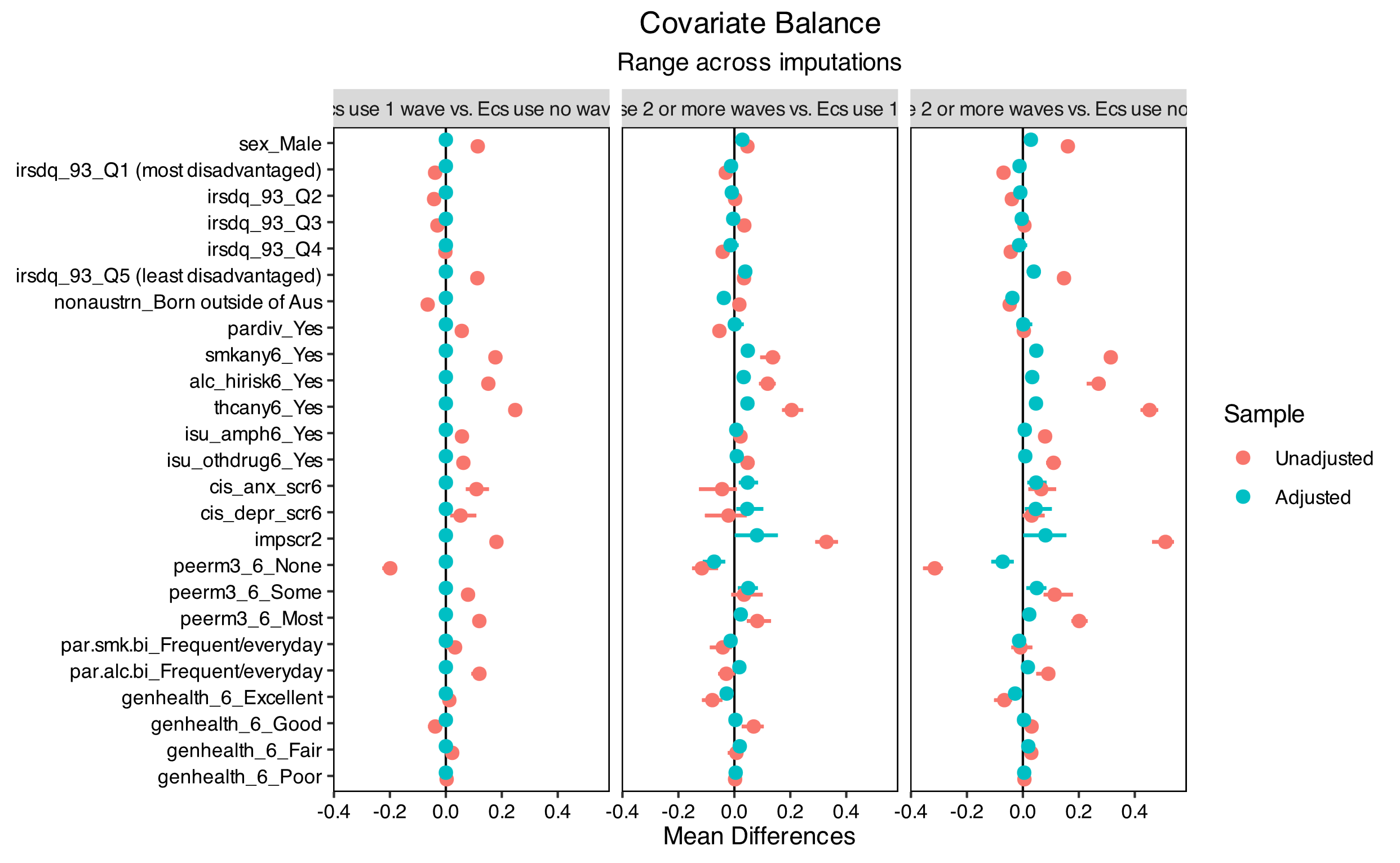


**Figure S11: Love plot displaying covariate balance for any persistent MDMA use exposure with weights truncated at 95^th^ percentile**


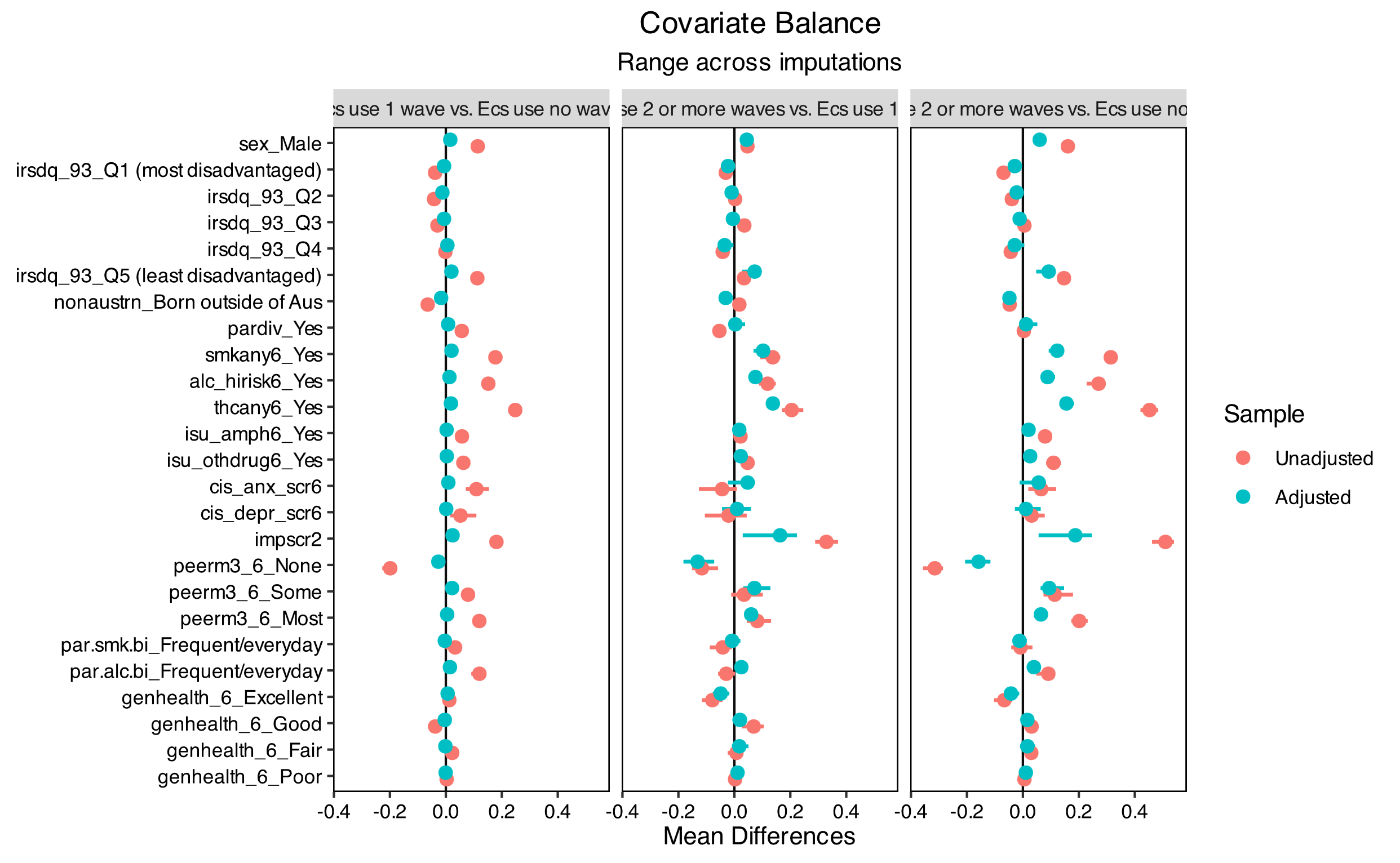


**Figure S12: Love plot displaying covariate balance for any persistent MDMA use exposure with weights truncated at 90^th^ percentile**


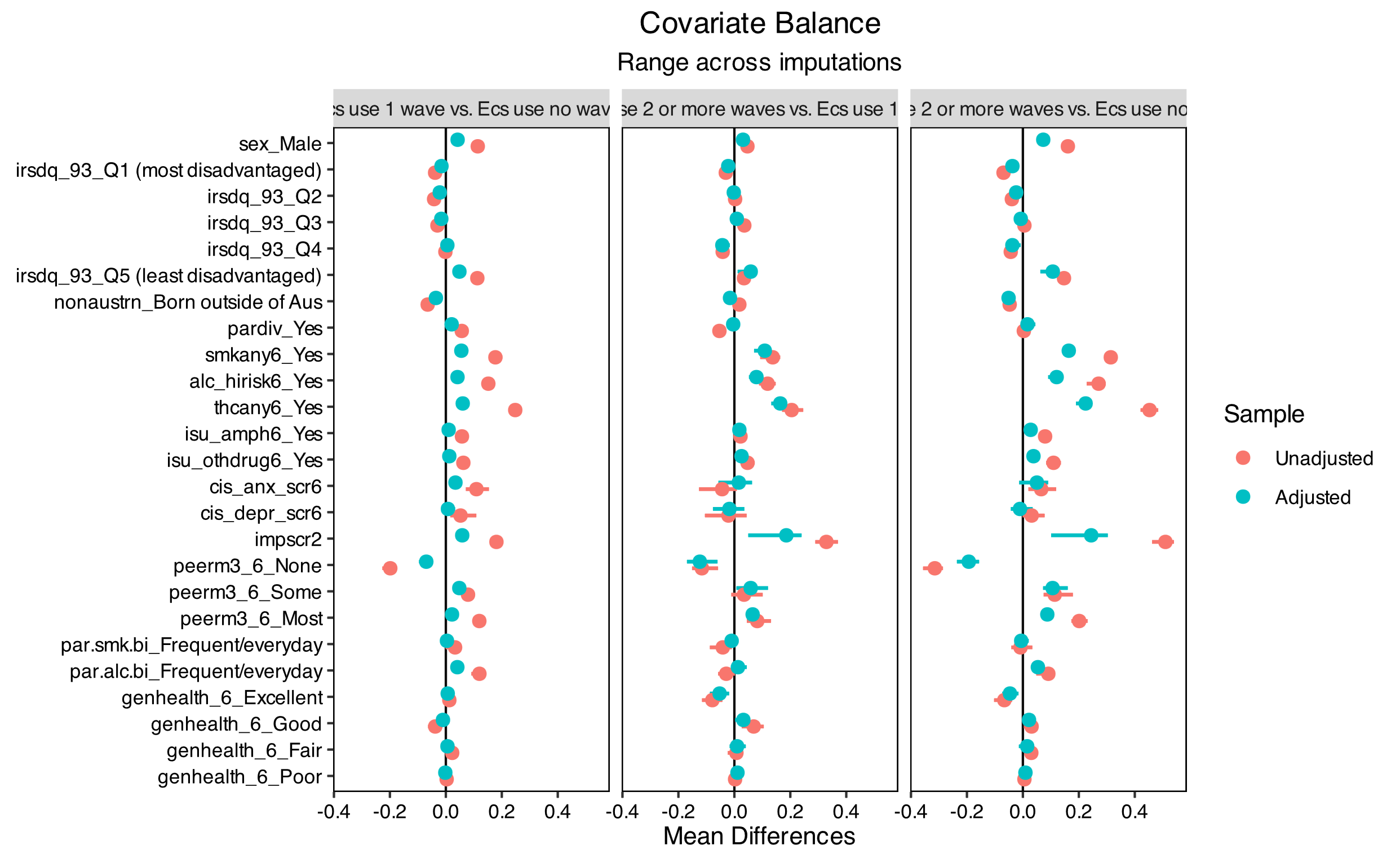


**Regression outcomes for wave 11 as outcome**

| **Table S8: Regression models for the relationship between any MDMA use and depression at wave 11** | | | | | | | | | |
| --- | --- | --- | --- | --- | --- | --- | --- | --- | --- |
|  | **Unadjusted model (n = 1329)** | | | **Imputed & adjusted model (n = 1329)** | | | **Doubly robust IPTW model (n = 1329)** | | |
| **Characteristic** | **OR** | **95% CI** | **p** | **OR** | **95% CI** | **p** | **OR** | **95% CI** | **p** |
| ***Any MDMA use*** |  |  |  |  |  |  |  |  |  |
| *No* | - | - | - | - | - | - | - | - | - |
| *Yes* | 0.93 | 0.64, 1.33 | 0.7 | 0.99 | 0.66, 1.48 | >0.9 | 1.04 | 0.68, 1.59 | 0.9 |
| ***Sex*** |  |  |  |  |  |  |  |  |  |
| *Female* |  |  |  | — | — |  | — | — |  |
| *Male* |  |  |  | 0.89 | 0.62, 1.28 | 0.5 | 0.8 | 0.52, 1.24 | 0.3 |
| ***Nationality*** |  |  |  |  |  |  |  |  |  |
| *Born in Aus* |  |  |  | — | — |  | — | — |  |
| *Born outside of Aus* |  |  |  | 0.86 | 0.50, 1.46 | 0.6 | 0.99 | 0.52, 1.87 | >0.9 |
| ***Socioeconomic disadvantage quintiles*** |  |  |  |  |  |  |  |  |  |
| *Q1 (most disadvantaged)* |  |  |  | — | — |  | — | — |  |
| *Q2* |  |  |  | 1.31 | 0.70, 2.45 | 0.4 | 1.36 | 0.60, 3.06 | 0.5 |
| *Q3* |  |  |  | 1.14 | 0.60, 2.17 | 0.7 | 1.06 | 0.47, 2.42 | 0.9 |
| *Q4* |  |  |  | 0.99 | 0.55, 1.78 | >0.9 | 0.72 | 0.35, 1.52 | 0.4 |
| *Q5 (least disadvantaged)* |  |  |  | 0.94 | 0.54, 1.62 | 0.8 | 0.7 | 0.35, 1.41 | 0.3 |
| ***Parental divorce*** |  |  |  |  |  |  |  |  |  |
| *No* |  |  |  | — | — |  | — | — |  |
| *Yes* |  |  |  | 1.2 | 0.79, 1.81 | 0.4 | 1.54 | 0.92, 2.58 | 0.1 |
| ***General health*** |  |  |  |  |  |  |  |  |  |
| *Excellent* |  |  |  | — | — |  | — | — |  |
| *Good* |  |  |  | 0.86 | 0.54, 1.36 | 0.5 | 0.82 | 0.47, 1.42 | 0.5 |
| *Fair* |  |  |  | 1.13 | 0.64, 1.98 | 0.7 | 0.97 | 0.50, 1.88 | >0.9 |
| *Poor* |  |  |  | 2.07 | 0.76, 5.65 | 0.2 | 1.16 | 0.29, 4.61 | 0.8 |
| ***Any smoking*** |  |  |  |  |  |  |  |  |  |
| *No* |  |  |  | — | — |  | — | — |  |
| *Yes* |  |  |  | 1.15 | 0.73, 1.82 | 0.5 | 1.49 | 0.86, 2.57 | 0.2 |
| ***Any binge drinking*** |  |  |  |  |  |  |  |  |  |
| *No* |  |  |  | — | — |  | — | — |  |
| *Yes* |  |  |  | 0.86 | 0.51, 1.43 | 0.6 | 0.79 | 0.42, 1.47 | 0.5 |
| ***Any cannabis use*** |  |  |  |  |  |  |  |  |  |
| *No* |  |  |  | — | — |  | — | — |  |
| *Yes* |  |  |  | 1.07 | 0.59, 1.93 | 0.8 | 0.92 | 0.46, 1.82 | 0.8 |
| ***Any amphetamine use*** |  |  |  |  |  |  |  |  |  |
| *No* |  |  |  | — | — |  | — | — |  |
| *Yes* |  |  |  | 1.23 | 0.38, 3.96 | 0.7 | 1.22 | 0.27, 5.54 | 0.8 |
| ***Any other drug use*** |  |  |  |  |  |  |  |  |  |
| *No* |  |  |  | — | — |  | — | — |  |
| *Yes* |  |  |  | 0.83 | 0.30, 2.27 | 0.7 | 0.61 | 0.17, 2.17 | 0.4 |
| ***Peer substance use*** |  |  |  |  |  |  |  |  |  |
| *None* |  |  |  | — | — |  | — | — |  |
| *Some* |  |  |  | 0.7 | 0.45, 1.08 | 0.11 | 0.7 | 0.41, 1.20 | 0.2 |
| *Most* |  |  |  | 0.92 | 0.44, 1.94 | 0.8 | 1.01 | 0.40, 2.53 | >0.9 |
| ***Parental smoking*** |  |  |  |  |  |  |  |  |  |
| *Never/Infrequent* |  |  |  | — | — |  | — | — |  |
| *Frequent/everyday* |  |  |  | 1.28 | 0.87, 1.89 | 0.2 | 1.25 | 0.77, 2.01 | 0.4 |
| ***Parental alcohol use*** |  |  |  |  |  |  |  |  |  |
| *Never/Infrequent* |  |  |  | — | — |  | — | — |  |
| *Frequent/everyday* |  |  |  | 0.86 | 0.55, 1.33 | 0.5 | 0.76 | 0.46, 1.25 | 0.3 |
| ***Anxiety score at wave 6*** |  |  |  | 1.02 | 0.79, 1.33 | 0.9 | 1.04 | 0.79, 1.36 | 0.8 |
| ***Depression score at wave 6*** |  |  |  | 1.28 | 1.03, 1.58 | 0.026 | 1.31 | 1.01, 1.70 | 0.045 |
| *I****mpulsivity score*** |  |  |  | 0.99 | 0.93, 1.05 | 0.7 | 1.02 | 0.94, 1.10 | 0.7 |

| **Table S9: Regression models for the relationship between persistent MDMA use and depression at wave 11** | | | | | | | | | |
| --- | --- | --- | --- | --- | --- | --- | --- | --- | --- |
|  | **Unadjusted model (n = 1329)** | | | **Imputed & adjusted model (n = 1329)** | | | **Doubly robust IPTW model (n = 1329)** | | |
| **Characteristic** | **OR** | **95% CI** | **p** | **OR** | **95% CI** | **p** | **OR** | **95% CI** | **p** |
| ***Any persistent MDMA use*** |  |  |  |  |  |  |  |  |  |
| MDMA use no waves | - | - | - | - | - | - | - | - | - |
| MDMA use 1 wave | 0.67 | 0.39, 1.08 | 0.11 | 0.7 | 0.41, 1.19 | 0.2 | 1.02 | 0.65, 1.62 | >0.9 |
| MDMA use 2 or more waves | 1.32 | 0.81, 2.09 | 0.2 | 1.51 | 0.89, 2.54 | 0.13 | 2.17 | 1.30, 3.62 | **0.003** |
| ***Sex*** |  |  |  |  |  |  |  |  |  |
| *Female* |  |  |  | — | — |  | — | — |  |
| *Male* |  |  |  | 0.88 | 0.61, 1.27 | 0.5 | 0.76 | 0.48, 1.19 | 0.2 |
| ***Nationality*** |  |  |  |  |  |  |  |  |  |
| *Born in Aus* |  |  |  | — | — |  | — | — |  |
| *Born outside of Aus* |  |  |  | 0.85 | 0.50, 1.46 | 0.6 | 1.46 | 0.73, 2.92 | 0.3 |
| ***Socioeconomic disadvantage quintiles*** |  |  |  |  |  |  |  |  |  |
| *Q1 (most disadvantaged)* |  |  |  | — | — |  | — | — |  |
| *Q2* |  |  |  | 1.28 | 0.68, 2.39 | 0.4 | 0.73 | 0.31, 1.72 | 0.5 |
| *Q3* |  |  |  | 1.11 | 0.58, 2.11 | 0.8 | 0.85 | 0.34, 2.12 | 0.7 |
| *Q4* |  |  |  | 0.97 | 0.54, 1.75 | >0.9 | 0.94 | 0.43, 2.05 | 0.9 |
| *Q5 (least disadvantaged)* |  |  |  | 0.91 | 0.52, 1.57 | 0.7 | 1.04 | 0.51, 2.12 | >0.9 |
| ***Parental divorce*** |  |  |  |  |  |  |  |  |  |
| *No* |  |  |  | — | — |  | — | — |  |
| *Yes* |  |  |  | 1.22 | 0.81, 1.85 | 0.3 | 1.21 | 0.69, 2.13 | 0.5 |
| ***General health*** |  |  |  |  |  |  |  |  |  |
| *Excellent* |  |  |  | — | — |  | — | — |  |
| *Good* |  |  |  | 0.85 | 0.54, 1.35 | 0.5 | 0.85 | 0.46, 1.55 | 0.6 |
| *Fair* |  |  |  | 1.12 | 0.64, 1.98 | 0.7 | 0.78 | 0.39, 1.56 | 0.5 |
| *Poor* |  |  |  | 2.08 | 0.77, 5.65 | 0.15 | 6.04 | 1.57, 23.2 | **0.009** |
| ***Any smoking*** |  |  |  |  |  |  |  |  |  |
| *No* |  |  |  | — | — |  | — | — |  |
| *Yes* |  |  |  | 1.14 | 0.72, 1.82 | 0.6 | 1.64 | 0.90, 2.98 | 0.11 |
| ***Any binge drinking*** |  |  |  |  |  |  |  |  |  |
| *No* |  |  |  | — | — |  | — | — |  |
| *Yes* |  |  |  | 0.84 | 0.50, 1.41 | 0.5 | 0.7 | 0.38, 1.32 | 0.3 |
| ***Any cannabis use*** |  |  |  |  |  |  |  |  |  |
| *No* |  |  |  | — | — |  | — | — |  |
| *Yes* |  |  |  | 1.02 | 0.56, 1.86 | >0.9 | 0.73 | 0.37, 1.46 | 0.4 |
| ***Any amphetamine use*** |  |  |  |  |  |  |  |  |  |
| *No* |  |  |  | — | — |  | — | — |  |
| *Yes* |  |  |  | 1.28 | 0.40, 4.12 | 0.7 | 1.03 | 0.31, 3.39 | >0.9 |
| ***Any other drug use*** |  |  |  |  |  |  |  |  |  |
| *No* |  |  |  | — | — |  | — | — |  |
| *Yes* |  |  |  | 0.86 | 0.31, 2.36 | 0.8 | 2.02 | 0.77, 5.32 | 0.2 |
| ***Peer substance use*** |  |  |  |  |  |  |  |  |  |
| *None* |  |  |  | — | — |  | — | — |  |
| *Some* |  |  |  | 0.7 | 0.45, 1.09 | 0.12 | 0.76 | 0.42, 1.38 | 0.4 |
| *Most* |  |  |  | 0.92 | 0.44, 1.94 | 0.8 | 0.9 | 0.36, 2.26 | 0.8 |
| ***Parental smoking*** |  |  |  |  |  |  |  |  |  |
| *Never/Infrequent* |  |  |  | — | — |  | — | — |  |
| *Frequent/everyday* |  |  |  | 1.29 | 0.87, 1.90 | 0.2 | 1.11 | 0.69, 1.80 | 0.7 |
| ***Parental alcohol use*** |  |  |  |  |  |  |  |  |  |
| *Never/Infrequent* |  |  |  | — | — |  | — | — |  |
| *Frequent/everyday* |  |  |  | 0.86 | 0.55, 1.35 | 0.5 | 0.98 | 0.59, 1.61 | >0.9 |
| ***Anxiety score at wave 6*** |  |  |  | 1.03 | 0.79, 1.35 | 0.8 | 0.85 | 0.61, 1.20 | 0.4 |
| ***Depression score at wave 6*** |  |  |  | 1.27 | 1.02, 1.57 | **0.03** | 1.32 | 1.00, 1.75 | **0.053** |
| *I****mpulsivity score*** |  |  |  | 0.99 | 0.93, 1.04 | 0.6 | 1.07 | 0.99, 1.14 | **0.074** |

| **Table S10: Regression models for the relationship between any frequent MDMA use and depression wave 11** | | | | | | | | | |
| --- | --- | --- | --- | --- | --- | --- | --- | --- | --- |
|  | **Unadjusted model (n = 1221)** | | | **Imputed & adjusted model (n = 1221)** | | | **Doubly robust IPTW model (n = 1221)** | | |
| **Characteristic** | **OR** | **95% CI** | **p** | **OR** | **95% CI** | **p** | **OR** | **95% CI** | **p** |
| ***Any frequent MDMA use*** |  |  |  |  |  |  |  |  |  |
| Never | - | - | - | - | - | - | - | - | - |
| Infrequent/experimental | 1.14 | 0.71, 1.76 | 0.6 | 1.26 | 0.78, 2.05 | 0.3 | 1.41 | 0.82, 2.41 | 0.2 |
| Frequent | 1.06 | 0.48, 2.08 | 0.9 | 1.19 | 0.55, 2.57 | 0.7 | 1.28 | 0.55, 2.98 | 0.6 |
| ***Sex*** |  |  |  |  |  |  |  |  |  |
| *Female* |  |  |  | — | — |  | — | — |  |
| *Male* |  |  |  | 0.88 | 0.61, 1.26 | 0.5 | 0.83 | 0.47, 1.45 | 0.5 |
| ***Nationality*** |  |  |  |  |  |  |  |  |  |
| *Born in Aus* |  |  |  | — | — |  | — | — |  |
| *Born outside of Aus* |  |  |  | 0.85 | 0.50, 1.46 | 0.6 | 1.36 | 0.60, 3.06 | 0.5 |
| ***Socioeconomic disadvantage quintiles*** |  |  |  |  |  |  |  |  |  |
| *Q1 (most disadvantaged)* |  |  |  | — | — |  | — | — |  |
| *Q2* |  |  |  | 1.31 | 0.70, 2.46 | 0.4 | 0.99 | 0.36, 2.74 | >0.9 |
| *Q3* |  |  |  | 1.12 | 0.59, 2.14 | 0.7 | 0.68 | 0.25, 1.84 | 0.4 |
| *Q4* |  |  |  | 0.98 | 0.55, 1.77 | >0.9 | 0.4 | 0.15, 1.02 | **0.055** |
| *Q5 (least disadvantaged)* |  |  |  | 0.93 | 0.54, 1.60 | 0.8 | 0.43 | 0.18, 1.01 | **0.053** |
| ***Parental divorce*** |  |  |  |  |  |  |  |  |  |
| *No* |  |  |  | — | — |  | — | — |  |
| *Yes* |  |  |  | 1.21 | 0.80, 1.83 | 0.4 | 1.88 | 1.00, 3.55 | **0.052** |
| ***General health*** |  |  |  |  |  |  |  |  |  |
| *Excellent* |  |  |  | — | — |  | — | — |  |
| *Good* |  |  |  | 0.85 | 0.54, 1.36 | 0.5 | 0.73 | 0.37, 1.45 | 0.4 |
| *Fair* |  |  |  | 1.13 | 0.64, 1.98 | 0.7 | 0.95 | 0.40, 2.25 | >0.9 |
| *Poor* |  |  |  | 2.11 | 0.78, 5.73 | 0.14 | 4.31 | 0.77, 24.0 | 0.1 |
| ***Any smoking*** |  |  |  |  |  |  |  |  |  |
| *No* |  |  |  | — | — |  | — | — |  |
| *Yes* |  |  |  | 1.14 | 0.72, 1.81 | 0.6 | 1.77 | 0.88, 3.52 | 0.11 |
| ***Any binge drinking*** |  |  |  |  |  |  |  |  |  |
| *No* |  |  |  | — | — |  | — | — |  |
| *Yes* |  |  |  | 0.85 | 0.51, 1.42 | 0.5 | 0.85 | 0.38, 1.89 | 0.7 |
| ***Any cannabis use*** |  |  |  |  |  |  |  |  |  |
| *No* |  |  |  | — | — |  | — | — |  |
| *Yes* |  |  |  | 1.03 | 0.57, 1.86 | >0.9 | 0.89 | 0.34, 2.32 | 0.8 |
| ***Any amphetamine use*** |  |  |  |  |  |  |  |  |  |
| *No* |  |  |  | — | — |  | — | — |  |
| *Yes* |  |  |  | 1.21 | 0.38, 3.87 | 0.7 | 0.54 | 0.13, 2.30 | 0.4 |
| ***Any other drug use*** |  |  |  |  |  |  |  |  |  |
| *No* |  |  |  | — | — |  | — | — |  |
| *Yes* |  |  |  | 0.81 | 0.30, 2.24 | 0.7 | 0.69 | 0.19, 2.50 | 0.6 |
| ***Peer substance use*** |  |  |  |  |  |  |  |  |  |
| *None* |  |  |  | — | — |  | — | — |  |
| *Some* |  |  |  | 0.69 | 0.44, 1.06 | 0.093 | 0.58 | 0.28, 1.20 | 0.14 |
| *Most* |  |  |  | 0.91 | 0.44, 1.91 | 0.8 | 0.99 | 0.26, 3.71 | >0.9 |
| ***Parental smoking*** |  |  |  |  |  |  |  |  |  |
| *Never/Infrequent* |  |  |  | — | — |  | — | — |  |
| *Frequent/everyday* |  |  |  | 1.29 | 0.87, 1.89 | 0.2 | 1.16 | 0.64, 2.12 | 0.6 |
| ***Parental alcohol use*** |  |  |  |  |  |  |  |  |  |
| *Never/Infrequent* |  |  |  | — | — |  | — | — |  |
| *Frequent/everyday* |  |  |  | 0.85 | 0.54, 1.32 | 0.5 | 0.64 | 0.35, 1.18 | 0.2 |
| ***Anxiety score at wave 6*** |  |  |  | 1.02 | 0.78, 1.33 | 0.9 | 1.08 | 0.75, 1.54 | 0.7 |
| ***Depression score at wave 6*** |  |  |  | 1.27 | 1.03, 1.58 | **0.027** | 1.36 | 0.96, 1.93 | 0.085 |
| *I****mpulsivity score*** |  |  |  | 0.99 | 0.93, 1.05 | 0.7 | 1.03 | 0.94, 1.12 | 0.6 |

| **Table S11: Regression models for the relationship between any MDMA use and anxiety at wave 11** | | | | | | | | | |
| --- | --- | --- | --- | --- | --- | --- | --- | --- | --- |
|  | **Unadjusted model (n = 1329)** | | | **Imputed & adjusted model (n = 1329)** | | | **Doubly robust IPTW model (n = 1329)** | | |
| **Characteristic** | **OR** | **95% CI** | **p** | **OR** | **95% CI** | **p** | **OR** | **95% CI** | **p** |
| ***Any MDMA use*** |  |  |  |  |  |  |  |  |  |
| *No* | - | - | - | - | - | - | - | - | - |
| *Yes* | 1.34 | 0.98, 1.82 | 0.059 | 1.37 | 0.96, 1.95 | 0.08 | 1.35 | 0.94, 1.94 | 0.11 |
| ***Sex*** |  |  |  |  |  |  |  |  |  |
| *Female* |  |  |  | — | — |  | — | — |  |
| *Male* |  |  |  | 0.73 | 0.53, 1.01 | 0.061 | 0.78 | 0.53, 1.16 | 0.2 |
| ***Nationality*** |  |  |  |  |  |  |  |  |  |
| *Born in Aus* |  |  |  | — | — |  | — | — |  |
| *Born outside of Aus* |  |  |  | 0.97 | 0.60, 1.55 | 0.9 | 1.25 | 0.68, 2.31 | 0.5 |
| ***Socioeconomic disadvantage quintiles*** |  |  |  |  |  |  |  |  |  |
| *Q1 (most disadvantaged)* |  |  |  | — | — |  | — | — |  |
| *Q2* |  |  |  | 0.75 | 0.43, 1.34 | 0.3 | 0.8 | 0.39, 1.63 | 0.5 |
| *Q3* |  |  |  | 0.71 | 0.40, 1.26 | 0.2 | 0.9 | 0.42, 1.90 | 0.8 |
| *Q4* |  |  |  | 0.72 | 0.43, 1.20 | 0.2 | 0.89 | 0.46, 1.72 | 0.7 |
| *Q5 (least disadvantaged)* |  |  |  | 0.81 | 0.51, 1.28 | 0.4 | 1.05 | 0.58, 1.89 | 0.9 |
| ***Parental divorce*** |  |  |  |  |  |  |  |  |  |
| *No* |  |  |  | — | — |  | — | — |  |
| *Yes* |  |  |  | 1.11 | 0.77, 1.62 | 0.6 | 1.17 | 0.73, 1.89 | 0.5 |
| ***General health*** |  |  |  |  |  |  |  |  |  |
| *Excellent* |  |  |  | — | — |  | — | — |  |
| *Good* |  |  |  | 0.95 | 0.63, 1.43 | 0.8 | 0.87 | 0.53, 1.44 | 0.6 |
| *Fair* |  |  |  | 0.97 | 0.59, 1.59 | >0.9 | 0.87 | 0.48, 1.57 | 0.6 |
| *Poor* |  |  |  | 2.53 | 0.99, 6.49 | **0.053** | 3.86 | 1.25, 11.9 | 0.019 |
| ***Any smoking*** |  |  |  |  |  |  |  |  |  |
| *No* |  |  |  | — | — |  | — | — |  |
| *Yes* |  |  |  | 1.51 | 1.01, 2.25 | **0.045** | 1.51 | 0.92, 2.47 | 0.1 |
| ***Any binge drinking*** |  |  |  |  |  |  |  |  |  |
| *No* |  |  |  | — | — |  | — | — |  |
| *Yes* |  |  |  | 0.87 | 0.55, 1.37 | 0.6 | 0.8 | 0.47, 1.37 | 0.4 |
| ***Any cannabis use*** |  |  |  |  |  |  |  |  |  |
| *No* |  |  |  | — | — |  | — | — |  |
| *Yes* |  |  |  | 0.87 | 0.51, 1.49 | 0.6 | 0.88 | 0.48, 1.61 | 0.7 |
| ***Any amphetamine use*** |  |  |  |  |  |  |  |  |  |
| *No* |  |  |  | — | — |  | — | — |  |
| *Yes* |  |  |  | 1.21 | 0.44, 3.32 | 0.7 | 1.09 | 0.35, 3.37 | 0.9 |
| ***Any other drug use*** |  |  |  |  |  |  |  |  |  |
| *No* |  |  |  | — | — |  | — | — |  |
| *Yes* |  |  |  | 1.33 | 0.61, 2.91 | 0.5 | 1.49 | 0.60, 3.73 | 0.4 |
| ***Peer substance use*** |  |  |  |  |  |  |  |  |  |
| *None* |  |  |  | — | — |  | — | — |  |
| *Some* |  |  |  | 0.88 | 0.61, 1.29 | 0.5 | 0.75 | 0.46, 1.23 | 0.3 |
| *Most* |  |  |  | 1 | 0.50, 1.97 | >0.9 | 0.92 | 0.41, 2.04 | 0.8 |
| ***Parental smoking*** |  |  |  |  |  |  |  |  |  |
| *Never/Infrequent* |  |  |  | — | — |  | — | — |  |
| *Frequent/everyday* |  |  |  | 1.21 | 0.86, 1.69 | 0.3 | 1.09 | 0.73, 1.63 | 0.7 |
| ***Parental alcohol use*** |  |  |  |  |  |  |  |  |  |
| *Never/Infrequent* |  |  |  | — | — |  | — | — |  |
| *Frequent/everyday* |  |  |  | 0.76 | 0.52, 1.10 | 0.14 | 0.85 | 0.55, 1.31 | 0.5 |
| ***Anxiety score at wave 6*** |  |  |  | 1.09 | 0.86, 1.38 | 0.5 | 0.94 | 0.70, 1.26 | 0.7 |
| ***Depression score at wave 6*** |  |  |  | 1.13 | 0.92, 1.39 | 0.2 | 1.24 | 0.96, 1.61 | 0.1 |
| *I****mpulsivity score*** |  |  |  | 1 | 0.96, 1.05 | 0.9 | 1.05 | 0.98, 1.11 | 0.14 |

| **Table S12: Regression models for the relationship between persistent MDMA use and anxiety at wave 11** | | | | | | | | | |
| --- | --- | --- | --- | --- | --- | --- | --- | --- | --- |
|  | **Unadjusted model (n = 1329)** | | | **Imputed & adjusted model (n = 1329)** | | | **Doubly robust IPTW model (n = 1329)** | | |
| **Characteristic** | **OR** | **95% CI** | **p** | **OR** | **95% CI** | **p** | **OR** | **95% CI** | **p** |
| ***Any persistent MDMA use*** |  |  |  |  |  |  |  |  |  |
| MDMA use no waves | - | - | - | - | - | - | - | - | - |
| MDMA use 1 wave | 1.11 | 0.74, 1.63 | 0.6 | 1.11 | 0.72, 1.70 | 0.6 | 1.02 | 0.65, 1.62 | >0.9 |
| MDMA use 2 or more waves | 1.7 | 1.12, 2.55 | **0.011** | 1.84 | 1.16, 2.93 | **0.01** | 2.17 | 1.30, 3.62 | **0.003** |
| ***Sex*** |  |  |  |  |  |  |  |  |  |
| *Female* |  |  |  | — | — |  | — | — |  |
| *Male* |  |  |  | 0.73 | 0.53, 1.01 | **0.055** | 0.76 | 0.48, 1.19 | 0.2 |
| ***Nationality*** |  |  |  |  |  |  |  |  |  |
| *Born in Aus* |  |  |  | — | — |  | — | — |  |
| *Born outside of Aus* |  |  |  | 0.96 | 0.60, 1.54 | 0.9 | 1.46 | 0.73, 2.92 | 0.3 |
| ***Socioeconomic disadvantage quintiles*** |  |  |  |  |  |  |  |  |  |
| *Q1 (most disadvantaged)* |  |  |  | — | — |  | — | — |  |
| *Q2* |  |  |  | 0.74 | 0.42, 1.32 | 0.3 | 0.73 | 0.31, 1.72 | 0.5 |
| *Q3* |  |  |  | 0.69 | 0.39, 1.23 | 0.2 | 0.85 | 0.34, 2.12 | 0.7 |
| *Q4* |  |  |  | 0.71 | 0.43, 1.18 | 0.2 | 0.94 | 0.43, 2.05 | 0.9 |
| *Q5 (least disadvantaged)* |  |  |  | 0.79 | 0.50, 1.25 | 0.3 | 1.04 | 0.51, 2.12 | >0.9 |
| ***Parental divorce*** |  |  |  |  |  |  |  |  |  |
| *No* |  |  |  | — | — |  | — | — |  |
| *Yes* |  |  |  | 1.13 | 0.77, 1.64 | 0.5 | 1.21 | 0.69, 2.13 | 0.5 |
| ***General health*** |  |  |  |  |  |  |  |  |  |
| *Excellent* |  |  |  | — | — |  | — | — |  |
| *Good* |  |  |  | 0.94 | 0.62, 1.42 | 0.8 | 0.85 | 0.46, 1.55 | 0.6 |
| *Fair* |  |  |  | 0.96 | 0.59, 1.58 | 0.9 | 0.78 | 0.39, 1.56 | 0.5 |
| *Poor* |  |  |  | 2.52 | 0.98, 6.44 | 0.054 | 6.04 | 1.57, 23.2 | **0.009** |
| ***Any smoking*** |  |  |  |  |  |  |  |  |  |
| *No* |  |  |  | — | — |  | — | — |  |
| *Yes* |  |  |  | 1.51 | 1.01, 2.26 | 0.047 | 1.64 | 0.90, 2.98 | 0.11 |
| ***Any binge drinking*** |  |  |  |  |  |  |  |  |  |
| *No* |  |  |  | — | — |  | — | — |  |
| *Yes* |  |  |  | 0.85 | 0.54, 1.35 | 0.5 | 0.7 | 0.38, 1.32 | 0.3 |
| ***Any cannabis use*** |  |  |  |  |  |  |  |  |  |
| *No* |  |  |  | — | — |  | — | — |  |
| *Yes* |  |  |  | 0.84 | 0.49, 1.45 | 0.5 | 0.73 | 0.37, 1.46 | 0.4 |
| ***Any amphetamine use*** |  |  |  |  |  |  |  |  |  |
| *No* |  |  |  | — | — |  | — | — |  |
| *Yes* |  |  |  | 1.24 | 0.45, 3.42 | 0.7 | 1.03 | 0.31, 3.39 | >0.9 |
| ***Any other drug use*** |  |  |  |  |  |  |  |  |  |
| *No* |  |  |  | — | — |  | — | — |  |
| *Yes* |  |  |  | 1.37 | 0.63, 2.99 | 0.4 | 2.02 | 0.77, 5.32 | 0.2 |
| ***Peer substance use*** |  |  |  |  |  |  |  |  |  |
| *None* |  |  |  | — | — |  | — | — |  |
| *Some* |  |  |  | 0.89 | 0.61, 1.30 | 0.5 | 0.76 | 0.42, 1.38 | 0.4 |
| *Most* |  |  |  | 1 | 0.51, 1.97 | >0.9 | 0.9 | 0.36, 2.26 | 0.8 |
| ***Parental smoking*** |  |  |  |  |  |  |  |  |  |
| *Never/Infrequent* |  |  |  | — | — |  | — | — |  |
| *Frequent/everyday* |  |  |  | 1.21 | 0.87, 1.69 | 0.3 | 1.11 | 0.69, 1.80 | 0.7 |
| ***Parental alcohol use*** |  |  |  |  |  |  |  |  |  |
| *Never/Infrequent* |  |  |  | — | — |  | — | — |  |
| *Frequent/everyday* |  |  |  | 0.76 | 0.53, 1.11 | 0.2 | 0.98 | 0.59, 1.61 | >0.9 |
| ***Anxiety score at wave 6*** |  |  |  | 1.09 | 0.86, 1.39 | 0.5 | 0.85 | 0.61, 1.20 | 0.4 |
| ***Depression score at wave 6*** |  |  |  | 1.13 | 0.92, 1.39 | 0.3 | 1.32 | 1.00, 1.75 | **0.053** |
| *I****mpulsivity score*** |  |  |  | 1 | 0.95, 1.05 | >0.9 | 1.07 | 0.99, 1.14 | 0.074 |

| **Table S13: Regression models for the relationship between any frequent MDMA use and anxiety at wave 11** | | | | | | | | | |
| --- | --- | --- | --- | --- | --- | --- | --- | --- | --- |
|  | **Unadjusted model (n = 1329)** | | | **Imputed & adjusted model (n = 1329)** | | | **Doubly robust IPTW model (n = 1329)** | | |
| **Characteristic** | **OR** | **95% CI** | **p** | **OR** | **95% CI** | **p** | **OR** | **95% CI** | **p** |
| ***Any frequent MDMA use*** |  |  |  |  |  |  |  |  |  |
| Never | - | - | - | - | - | - | - | - | - |
| Infrequent/experimental | 1.66 | 1.13, 2.41 | 0.008 | 1.74 | 1.15, 2.62 | 0.009 | 1.79 | 1.12, 2.86 | **0.015** |
| Frequent | 1.31 | 0.69, 2.36 | 0.4 | 1.41 | 0.73, 2.72 | 0.3 | 1.46 | 0.73, 2.91 | 0.3 |
| ***Sex*** |  |  |  |  |  |  |  |  |  |
| *Female* |  |  |  | — | — |  | — | — |  |
| *Male* |  |  |  | 0.73 | 0.52, 1.00 | 0.052 | 0.72 | 0.43, 1.21 | 0.2 |
| ***Nationality*** |  |  |  |  |  |  |  |  |  |
| *Born in Aus* |  |  |  | — | — |  | — | — |  |
| *Born outside of Aus* |  |  |  | 0.95 | 0.60, 1.53 | 0.8 | 1.26 | 0.58, 2.73 | 0.6 |
| ***Socioeconomic disadvantage quintiles*** |  |  |  |  |  |  |  |  |  |
| *Q1 (most disadvantaged)* |  |  |  | — | — |  | — | — |  |
| *Q2* |  |  |  | 0.76 | 0.43, 1.34 | 0.3 | 0.66 | 0.24, 1.76 | 0.4 |
| *Q3* |  |  |  | 0.7 | 0.39, 1.24 | 0.2 | 0.58 | 0.21, 1.58 | 0.3 |
| *Q4* |  |  |  | 0.71 | 0.43, 1.18 | 0.2 | 0.9 | 0.37, 2.21 | 0.8 |
| *Q5 (least disadvantaged)* |  |  |  | 0.8 | 0.50, 1.26 | 0.3 | 1.04 | 0.46, 2.37 | >0.9 |
| ***Parental divorce*** |  |  |  |  |  |  |  |  |  |
| *No* |  |  |  | — | — |  | — | — |  |
| *Yes* |  |  |  | 1.13 | 0.77, 1.64 | 0.5 | 1.38 | 0.75, 2.52 | 0.3 |
| ***General health*** |  |  |  |  |  |  |  |  |  |
| *Excellent* |  |  |  | — | — |  | — | — |  |
| *Good* |  |  |  | 0.94 | 0.63, 1.42 | 0.8 | 0.83 | 0.43, 1.60 | 0.6 |
| *Fair* |  |  |  | 0.97 | 0.59, 1.58 | 0.9 | 0.83 | 0.40, 1.75 | 0.6 |
| *Poor* |  |  |  | 2.58 | 1.00, 6.61 | 0.049 | 6.68 | 1.19, 37.5 | **0.03** |
| ***Any smoking*** |  |  |  |  |  |  |  |  |  |
| *No* |  |  |  | — | — |  | — | — |  |
| *Yes* |  |  |  | 1.5 | 1.00, 2.24 | 0.05 | 1.23 | 0.63, 2.39 | 0.5 |
| ***Any binge drinking*** |  |  |  |  |  |  |  |  |  |
| *No* |  |  |  | — | — |  | — | — |  |
| *Yes* |  |  |  | 0.87 | 0.55, 1.37 | 0.6 | 0.88 | 0.45, 1.71 | 0.7 |
| ***Any cannabis use*** |  |  |  |  |  |  |  |  |  |
| *No* |  |  |  | — | — |  | — | — |  |
| *Yes* |  |  |  | 0.85 | 0.50, 1.46 | 0.6 | 0.96 | 0.44, 2.12 | >0.9 |
| ***Any amphetamine use*** |  |  |  |  |  |  |  |  |  |
| *No* |  |  |  | — | — |  | — | — |  |
| *Yes* |  |  |  | 1.23 | 0.45, 3.37 | 0.7 | 0.88 | 0.29, 2.70 | 0.8 |
| ***Any other drug use*** |  |  |  |  |  |  |  |  |  |
| *No* |  |  |  | — | — |  | — | — |  |
| *Yes* |  |  |  | 1.32 | 0.61, 2.89 | 0.5 | 2.04 | 0.73, 5.73 | 0.2 |
| ***Peer substance use*** |  |  |  |  |  |  |  |  |  |
| *None* |  |  |  | — | — |  | — | — |  |
| *Some* |  |  |  | 0.88 | 0.60, 1.28 | 0.5 | 0.67 | 0.35, 1.26 | 0.2 |
| *Most* |  |  |  | 1.02 | 0.52, 2.00 | >0.9 | 0.82 | 0.29, 2.33 | 0.7 |
| ***Parental smoking*** |  |  |  |  |  |  |  |  |  |
| *Never/Infrequent* |  |  |  | — | — |  | — | — |  |
| *Frequent/everyday* |  |  |  | 1.21 | 0.87, 1.70 | 0.3 | 0.91 | 0.54, 1.54 | 0.7 |
| ***Parental alcohol use*** |  |  |  |  |  |  |  |  |  |
| *Never/Infrequent* |  |  |  | — | — |  | — | — |  |
| *Frequent/everyday* |  |  |  | 0.76 | 0.52, 1.09 | 0.14 | 0.91 | 0.53, 1.55 | 0.7 |
| ***Anxiety score at wave 6*** |  |  |  | 1.08 | 0.85, 1.38 | 0.5 | 0.83 | 0.55, 1.25 | 0.4 |
| ***Depression score at wave 6*** |  |  |  | 1.12 | 0.91, 1.38 | 0.3 | 1.32 | 0.93, 1.87 | 0.12 |
| *I****mpulsivity score*** |  |  |  | 1 | 0.95, 1.05 | >0.9 | 1.04 | 0.97, 1.12 | 0.2 |

**E-value sensitivity analysis**

| **Table S13: E-values** | | | | | |
| --- | --- | --- | --- | --- | --- |
| **Exposure** | **OR** | **95% CI** | **E-value (point)** | **E-value (lower CI)** | **Robustness** |
| Any MDMA use – Yes | 1.73 | 1.12-2.68 | 2.85 | 1.49 | Moderate |
| Any persistent MDMA use – 2+ waves | 2.05 | 1.07-3.94 | 3.52 | 1.34 | Moderate-strong |
| Any frequent MDMA use - Infrequent/experimental | 2.11 | 1.14-3.92 | 3.64 | 1.54 | Moderate-strong |
| Any frequent MDMA use – Frequent | 2.56 | 1.15-5.71 | 4.56 | 1.57 | Strong |

The E-values below indicate that sizeable unmeasured confounding would be required to fully explain away the observed associations, particularly for persistent and frequent MDMA use. For example, the E-value for frequent MDMA use (OR = 2.56) was 4.56, suggesting that a confounder would need to be associated with both MDMA use and risk of anxiety diagnosis by a odds ratio of over 4.5 each to nullify the finding. While more modest confounding (RR ≈ 1.5) could attenuate the confidence intervals, the associations generally appear moderately to strongly robust to unmeasured confounding (3).

| **Table S14. Early anxiety and depressive symptoms predicting later diagnosis** | | | | |
| --- | --- | --- | --- | --- |
| **Exposure** | **Outcome** | **OR** | **95% CI** | **P-value** |
| Depressive symptoms – wave 6 | Depression diagnosis – wave 10 | 1.28 | 1.03, 1.57 | 0.02 |
| Depressive symptoms – wave 6 | Anxiety diagnosis – wave 10 | 1.40 | 1.13, 1.71 | 0.001 |
| Anxiety symptoms – wave 6 | Anxiety diagnosis – wave 10 | 1.50 | 1.19, 1.86 | <0.001 |
| Anxiety symptoms – wave 6 | Depression diagnosis – wave 10 | 1.13 | 1.04, 1.65 | 0.02 |

**VAHCS study recruitment details**

The VAHCS study is a long-standing population-based longitudinal study commencing in 1992 as a statewide representative sample of Year 9 students (14-15 years old) from secondary schools across Victoria, Australia.

Participants were recruited using a two-stage cluster sampling procedure. During stage one, 45 schools that had multiple classes per level were randomly selected with a probability proportional to the number of year 9 students in the schools in each stratum. During the second stage, we randomly selected one class from each school at the end of year 9 (i.e., wave 1), and 6-months later we selected a second class from each school (i.e., wave 2). As a result, we had a close to representative cohort of Victorian year 10 students in 1992. After wave 1, one school did not continue (n=13 students), meaning that 44 schools with 2032 students were recruited. Of these students, 1943 (96%) participants in VAHCS at least once during waves 1-6.

Participants were followed up 11 times across three decades, with assessments during adolescence (ages 14-17), young adulthood (~ages 21 and 24), and adulthood (~ages 29, 35, and 41). The most recent wave (Wave 11) conducted in 2019-2021, in which 73% of the cohort was retained.

Before the students were invited to participate, we obtained written informed consent from the participants parent. Thereafter, adolescent consent was implied by their voluntary participation in study questionnaires. Following wave 6, adult participants gave their informed consent for each wave of participation (waves 7-11). The Human Research Ethics Committee of the Royal Children’s Hospital, Melbourne approved all data collection protocols.

Data from the adolescent phase (wave 1-6) were collected with in-school computer-administered questionnaires, data from the adult phase were collected with computer-assisted telephone interviews (waves 7-11) and online questionnaires (waves 10-11).

**Figure S1. Prevalence of MDMA use in the VAHCS cohort**


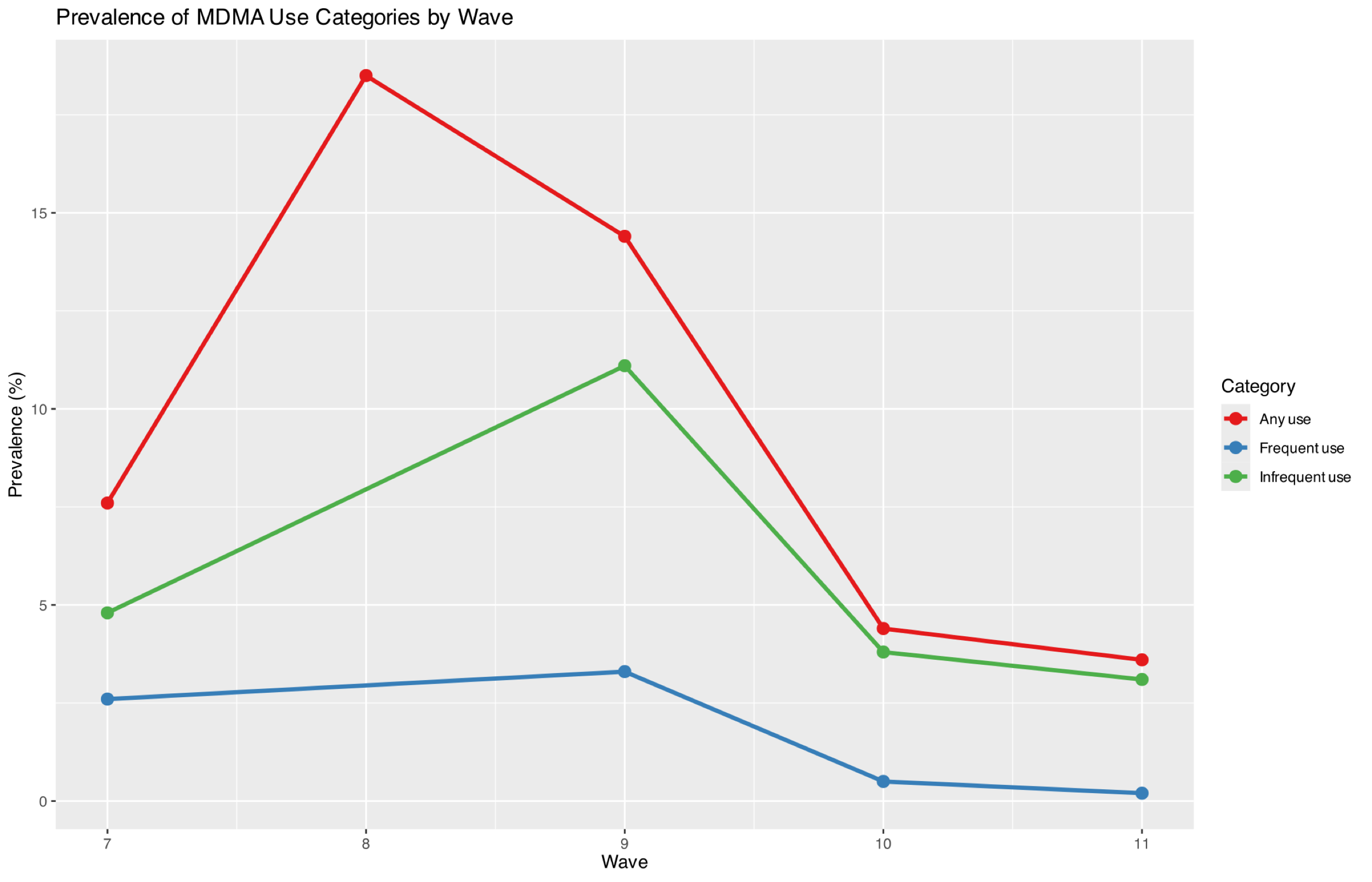


**References**

1. Thoemmes F, Ong AD. A Primer on Inverse Probability of Treatment Weighting and Marginal Structural Models. Emerging Adulthood. 2016;4(1).

2. Chesnaye NC, Stel VS, Tripepi G, Dekker FW, Fu EL, Zoccali C, et al. An introduction to inverse probability of treatment weighting in observational research. Vol. 15, Clinical Kidney Journal. 2022.

3. Van Der Weele TJ, Ding P. Sensitivity analysis in observational research: Introducing the E-Value. Ann Intern Med. 2017;167(4).
